# Genetic variation in *GNB5* causes bradycardia by augmenting the cholinergic response via increased acetylcholine-activated potassium current (*I*_K,ACh_)

**DOI:** 10.1242/dmm.037994

**Published:** 2019-07-09

**Authors:** Christiaan C. Veerman, Isabella Mengarelli, Charlotte D. Koopman, Ronald Wilders, Shirley C. van Amersfoorth, Diane Bakker, Rianne Wolswinkel, Mariam Hababa, Teun P. de Boer, Kaomei Guan, James Milnes, Elisabeth M. Lodder, Jeroen Bakkers, Arie O. Verkerk, Connie R. Bezzina

**Affiliations:** 1 Amsterdam UMC, University of Amsterdam, Department of Experimental Cardiology, Heart Center, 1105 AZ Amsterdam, The Netherlands; 2 Department of Medical Physiology, University Medical Center Utrecht, 3584 CX Utrecht, The Netherlands; 3 Hubrecht Institute, 3584 CT Utrecht, The Netherlands; 4 Amsterdam UMC, University of Amsterdam, Department of Medical Biology, Heart Failure Research Center, 1105 AZ Amsterdam, The Netherlands; 5 Department of Pharmacology and Toxicology, Technische Universität Dresden, 01062 Dresden, Germany; 6 Xention Ltd, Cambridge CB22 3EG, UK

**Keywords:** Electrophysiology, Mechanisms, Ion channels, Membrane transport, Transgenic models, Treatment, I(KACh)

## Abstract

Mutations in *GNB5*, encoding the G-protein β5 subunit (Gβ5), have recently been linked to a multisystem disorder that includes severe bradycardia. Here, we investigated the mechanism underlying bradycardia caused by the recessive p.S81L Gβ5 variant. Using CRISPR/Cas9-based targeting, we generated an isogenic series of human induced pluripotent stem cell (hiPSC) lines that were either wild type, heterozygous or homozygous for the *GNB5* p.S81L variant. These were differentiated into cardiomyocytes (hiPSC-CMs) that robustly expressed the acetylcholine-activated potassium channel [I(KACh); also known as I_K,ACh_]. Baseline electrophysiological properties of the lines did not differ. Upon application of carbachol (CCh), homozygous p.S81L hiPSC-CMs displayed an increased acetylcholine-activated potassium current (*I*_K,ACh_) density and a more pronounced decrease of spontaneous activity as compared to wild-type and heterozygous p.S81L hiPSC-CMs, explaining the bradycardia in homozygous carriers. Application of the specific I(KACh) blocker XEN-R0703 resulted in near-complete reversal of the phenotype. Our results provide mechanistic insights and proof of principle for potential therapy in patients carrying *GNB5* mutations.

This article has an associated First Person interview with the first author of the paper.

## Abstract (precis)

**Summary:** The authors show hiPSC-cardiomyocyte-based modeling of a mutation in a G-protein β-subunit that affects the electrophysiological response of cardiomyocytes to the cholinergic regulation of the heart rate, uncovering a potential therapy for severe bradycardia in humans carrying these mutations.

## INTRODUCTION

Inherited ion channel mutations are an important cause of sinoatrial node (SAN) dysfunction in the young (Verkerk and Wilders, 2015). The recent identification of rare genetic variants in genes encoding G-protein β (Gβ) subunits has expanded the repertoire of causal genes involved in SAN dysfunction beyond the previously described ion channel subunit genes (Lodder et al., 2016; Stallmeyer et al., 2017; Turkdogan et al., 2017; Vernon et al., 2018). Gβ subunits are components of heterotrimeric G-protein complexes that mediate G protein-coupled receptor signaling, which is involved in many processes, including slowing of heart rate (HR). Acetylcholine (ACh), released from post-ganglionic parasympathetic neurons, binds to M_2_ muscarinic receptors on pacemaker cells and atrial myocytes, triggering activation of heterotrimeric G-proteins that dissociate into Gα-GTP and Gβγ subunits. Of central importance is the effect of the Gβγ complex on the G protein-coupled inwardly rectifying K^+^ (GIRK) channel, which underlies the ACh-activated K^+^ channel [I(KACh); also known as I_K,ACh_]. I(KACh) is predominantly expressed in pacemaker cells and atrial myocytes (Dobrzynski et al., 2001) and is a heterotetramer consisting of Kir3.1 (encoded by *KCNJ3*) and Kir3.4 (encoded by *KCNJ5*) ion channel subunits (Krapivinsky et al., 1995). The Gβγ complex activates I(KACh) (Wickman et al., 1994), which, due to its permeability for K^+^ ions and inwardly rectifying properties (Sakmann et al., 1983), results in membrane potential hyperpolarization and slowing of diastolic depolarization in SAN cells, thereby decreasing spontaneous activity (DiFrancesco et al., 1989).

Recently, we and others reported mutations in *GNB5*, encoding Gβ5, as a cause of an autosomal recessive multisystem disorder including severe bradycardia at young age, necessitating pacemaker implantation (Lodder et al., 2016; Turkdogan et al., 2017; Vernon et al., 2018). As opposed to the other Gβ subunits, Gβ5 has an inhibitory effect on GIRK channels, thereby dampening the parasympathetic response, an effect that involves interaction with regulator of G-protein signaling (RGS) proteins (Lei et al., 2003; Wydeven et al., 2014). In patients carrying pathogenic variants in *GNB5*, the maximal HR during exercise is unaffected, whereas severe bradycardia occurs at rest, indicating a relation between the mutant *GNB5* and parasympathetic state (Lodder et al., 2016). Of the eight families described, the majority harbored recessive loss-of-function variants (i.e. nonsense, splice-site and frameshift) (Lodder et al., 2016; Turkdogan et al., 2017; Vernon et al., 2018), whereas the recessive missense variant c.242C>T p.S81L (NM_006578.3, rs761399728) was found in two independent families (Lodder et al., 2016). This missense variant is a low-frequency variant in individuals of Latin descent (minor allele frequency ∼3.2×10^−4^), where 1 in ∼1500 individuals is a heterozygous carrier (Lek et al., 2016).

The mechanism by which inherited genetic variation in *GNB5* causes excessive bradycardia remains unclear. By CRISPR/Cas9-based genome editing, we here generated an isogenic series of human induced pluripotent stem cell (hiPSC) lines (wild type, heterozygous and homozygous for Gβ5-S81L), which we differentiated into cardiomyocytes (hiPSC-CMs) that robustly express the I(KACh) channel by applying a retinoic acid (RA)-based protocol. We demonstrate that, in the homozygous state, the S81L variant results in an increased ACh-activated potassium current (*I*_K,ACh_) density and in excessive slowing of spontaneous activity upon stimulation with the cholinergic agonist carbachol (CCh). We also show reversibility of the phenotype by a specific blocker of I(KACh) (XEN-R0703), both in homozygous Gβ5-S81L hiPSC-CMs and *in vivo* in a zebrafish *gnb5*-knockout model, thus presenting proof of principle for pharmacological treatment of bradycardia as a consequence of genetic variants in *GNB5*.

## RESULTS

### Generation of Gβ5-S81L heterozygous and homozygous hiPSCs

To evaluate the electrophysiological consequences of the Gβ5-S81L variant, we introduced it into a control hiPSC line (Dudek et al., 2013) by means of CRISPR/Cas9-based genome editing, generating an isogenic series consisting of wild type, heterozygous and homozygous Gβ5-S81L hiPSC lines (hereafter annotated as WT, S81L^het^ and S81L^homo^, respectively) (Fig. S1A-C). All cell lines maintained expression of the pluripotency markers TRA1-81, OCT4, NANOG and SSEA4 (Fig. S1D-E, Table S2), exhibited a normal karyotype (Fig. S1F,G) and showed unmodified sequences at candidate gRNA off-target loci (Table S3). Data were generated from one clone per genotype. Relevant results were also confirmed in a second S81L^homo^ clone.

### Generation of hiPSC-CMs in the presence of RA results in functional *I*_K,ACh_

**Fig. 1.**
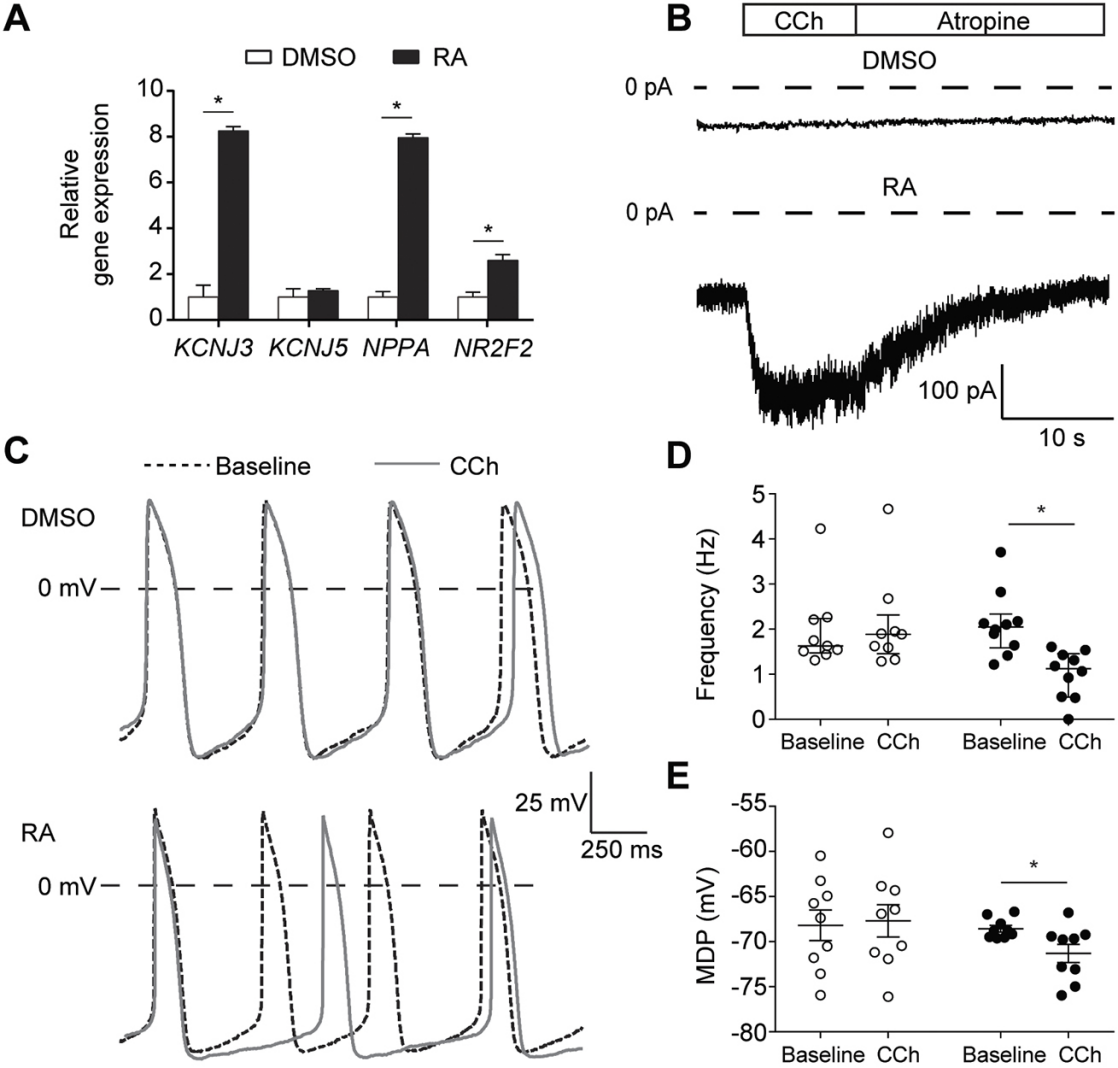
**Application of retinoic acid (RA) to differentiating hiPSCs results in cardiomyocytes (CMs) that exhibit a robust acetylcholine-activated K^+^ current (*I*_K,ACh_).** (A) Transcript expression of genes in RA-treated hiPSC-CMs relative to expression in DMSO-treated hiPSC-CMs. Data are mean±s.e.m. of three biological replicates, normalized to *TBP*. RA treatment enhanced expression of the genes *KCNJ3*, *NPPA* and *NR2F2*. *P<0.05 (two-sided *t*-tests). (B) Typical *I*_K,ACh_ traces in DMSO- and RA-treated hiPSC-CMs initiated by fast application of 100 µmol l^−1^ carbachol (CCh). After reaching steady state, addition of 1 mmol l^−1^ atropine achieves instant removal of CCh from muscarinic receptors and deactivation of the current. In DMSO-treated hiPSC-CMs, *I*_K,ACh_ was not detected, while RA-treated hiPSC-CMs all demonstrated a robust current (for average data, see Fig. 2). (C) Typical spontaneous action potentials (APs) of DMSO- and RA-treated hiPSC-CMs at baseline and upon addition of 10 µmol l^−1^ CCh. (D,E) Frequency of spontaneous APs (D: *n*=9, DMSO; *n*=10, RA) and maximal diastolic potential (MDP; E: *n*=9, DMSO; *n*=9, RA) of each cell at baseline and in the presence of CCh in DMSO- and RA-treated hiPSC-CMs. *P<0.05 (two-way ANOVA). CCh reduces the AP frequency and causes significant MDP hyperpolarization in RA-treated, but not in DMSO-treated, hiPSC-CMs.

To study the effect of the Gβ5-S81L variant on *I*_K,ACh_, we differentiated the three isogenic hiPSC lines into hiPSC-CMs using an RA-based differentiation protocol that promotes acquisition of atrial-like fate with robust expression of I(KACh). In line with our previous observations (Devalla et al., 2015; Marczenke et al., 2017), we found that, in WT hiPSC-CMs, the atria-enriched transcripts *NPPA* and *NR2F2* were upregulated upon RA treatment, as was the transcript of *KCNJ3*, encoding Kir3.1 (Fig. 1A). In both DMSO- and RA-treated WT hiPSC-CMs, we observed spontaneously active cells. Spontaneous activity is a consistent finding in hiPSC-CMs (Veerman et al., 2015) due to an (almost) complete lack of inwardly rectifying K^+^ current (*I*_K1_) (Meijer van Putten et al., 2015) and presence of the hyperpolarization-activated inward current (*I*_f_) (Ma et al., 2011) and ‘Ca^2+^ clock’ (Kim et al., 2015). For action potential (AP) measurements, we selected cells with a high beating rate (>1 Hz) to evaluate the effects of CCh on beating frequency and cells with a low beating rate (<1 Hz) to assess AP properties with and without CCh at a fixed pacing rate of 1 Hz. As reported previously (Marczenke et al., 2017), RA-treated WT hiPSC-CMs displayed shorter APs with a lower plateau as compared to DMSO-treated WT hiPSC-CMs, both during spontaneous activity and when paced at 1 Hz, thus showing a more atrial-like AP morphology (Fig. S2). In voltage-clamp experiments, we confirmed the presence of *I*_K,ACh_ in RA-treated WT hiPSC-CMs. By applying CCh, a robust current could be evoked in RA-treated and not in DMSO-treated WT hiPSC-CMs (Fig. 1B). This *I*_K,ACh_ deactivated upon rapid addition of the muscarinic antagonist atropine (Fig. 1B). Functional *I*_K,ACh_ effects on spontaneous activity were subsequently demonstrated by AP measurements in spontaneously fast-beating hiPSC-CMs. Figure 1C shows typical examples; effects on frequency and maximal diastolic potential (MDP) are summarized in Fig. 1D and E, respectively. In RA-treated hiPSC-CMs, but not in DMSO-treated hiPSC-CMs, application of CCh caused a significant slowing of spontaneous activity due to hyperpolarization of the MDP and a slower diastolic depolarization rate. Thus, RA-treated hiPSC-CMs display an atrial-like phenotype with functional presence of *I*_K,ACh_ in line with previous observations (Devalla et al., 2015; Marczenke et al., 2017), confirming that these cells are indeed suitable to study the electrophysiological effects of the Gβ5-S81L variant on *I*_K,ACh_.

**Fig. 2.**
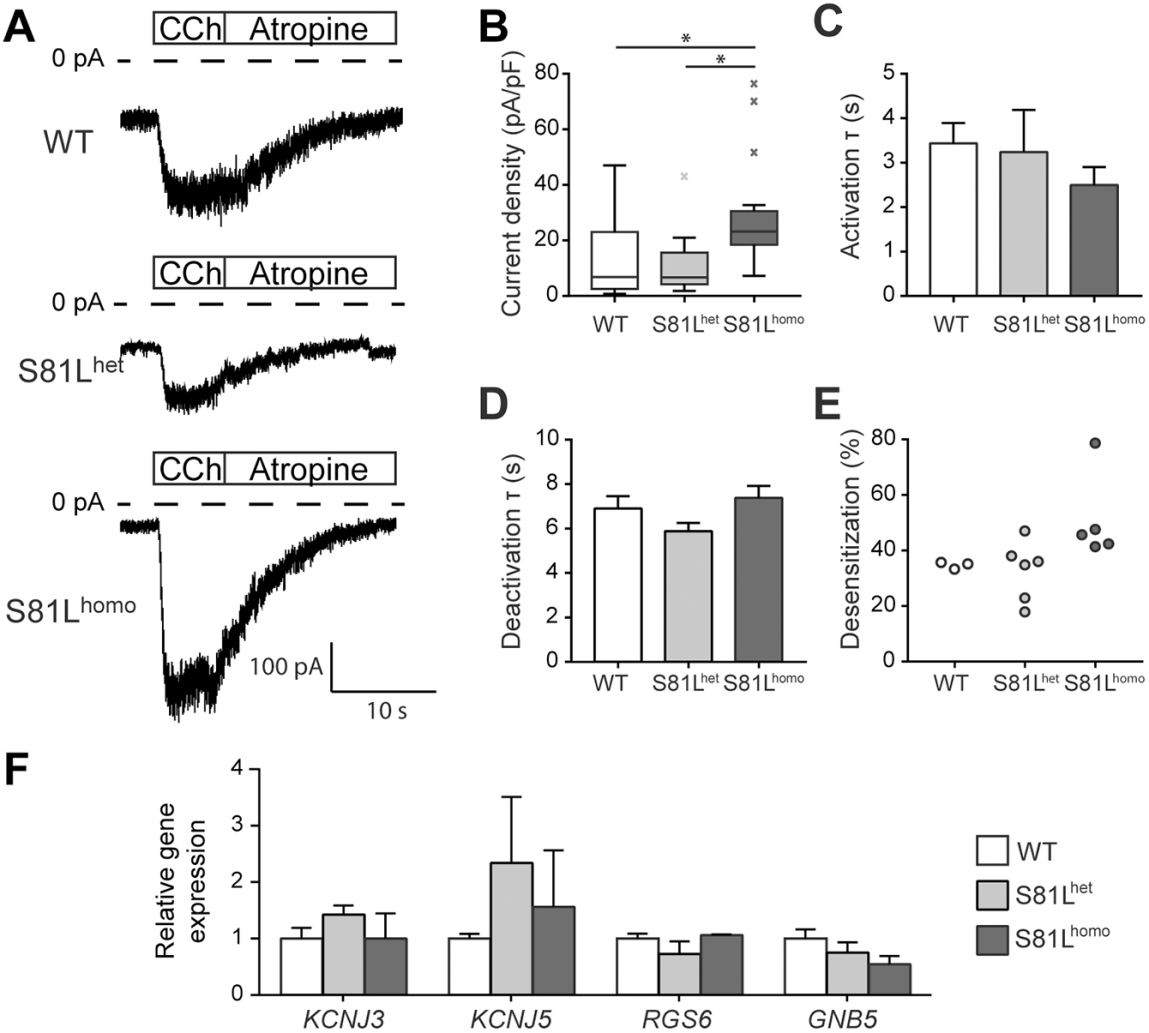
**Impact of the Gβ5-S81L variant on *I*_K,ACh_ in RA-treated hiPSC-CMs.** (A,B) Typical recordings (A) and average *I*_K,ACh_ density (B) in WT, S81L^het^ and S81L^homo^ hiPSC-CMs. Average *I*_K,ACh_ density is increased in S81L^homo^ hiPSC-CMs compared to WT and S81L^het^ hiPSC-CMs. *P<0.05 (Kruskal–Wallis non-parametric test, followed by Bonferroni post-hoc analysis). (C,D) Time constant (τ) of *I*_K,ACh_ activation (C) and deactivation (D). (B-D) WT, *n*=27; S81L^het^, *n*=11; S81L^homo^, *n*=20 hiPSC-CMs. (E) Percentage of desensitization, measured as the percentage of *I*_K,ACh_ decrease at steady state upon continuous CCh application; WT, *n*=3; S81L^het^, *n*=6; S81L^homo^, *n*=5 hiPSC-CMs. (F) Transcript level of the genes involved in G-protein-coupled activation of *I*_K,ACh_. Data are mean±s.e.m. of three biological differentiation replicas and expression level of each gene in S81L^het^ and S81L^homo^ hiPSC-CMs is indicated as relative to its expression in WT hiPSC-CMs. No significant differences are observed in *KCNJ3*, *KCNJ5*, *RGS6* and *GNB5* transcript abundance across the WT, S81L^het^ and S81L^homo^ hiPSC-CMs.

### *I*_K,ACh_ density is increased in S81L^homo^ hiPSC-CMs

Having established the presence and relevance of *I*_K,ACh_ in our RA-treated hiPSC-CM model, we next compared the properties of *I*_K,ACh_ in the isogenic WT, S81L^het^ and S81L^homo^ hiPSC-CMs. Figure 2A shows typical examples of *I*_K,ACh_ measurements. *I*_K,ACh_ density was significantly higher in S81L^homo^ hiPSC-CMs compared to S81L^het^ and WT (Fig. 2B). Moreover, activation and deactivation time constants were not significantly different between groups (Fig. 2C,D). In addition, desensitization was similar between groups (Fig. 2E). No significant differences were observed in *KCNJ3*, *KCNJ5*, *RGS6* and *GNB5* transcript abundance across the WT, S81L^het^ and S81L^homo^ lines, indicating that the increased *I*_K,ACh_ density in S81L^homo^ hiPSC-CMs was not due to altered expression of these genes (Fig. 2F). Thus, homozygous presence of the Gβ5-S81L variant increases *I*_K,ACh_ density without changes in transcript levels or kinetics of the channel. Additionally, to exclude differences in CM-cell population composition, we evaluated the expression level of a CM sarcomeric structural gene, *TNNT2*, in the hiPSC-CMs from the different lines; no significant variation in *TNNT2* expression was observed in hiPSC-CMs across the three different genotypes (Fig. S3). Also, no changes in the expression of *GNB1*, *GNB2* and *GNB4*, encoding the GIRK channel activatory β-subunits, was observed by reverse-transcription qPCR (RT-qPCR) (Fig. S3). The expression of *GNB3* could not be assessed due to large variation. These results indicate that the observed increase in *I*_K,ACh_ does not seem to be dependent on the upregulation of these activatory β-subunits in Gβ5-S81L hiPSC-CMs.

### S81L^homo^ hiPSC-CMs demonstrate a severe reduction in beating frequency upon CCh application

**Fig. 3.**
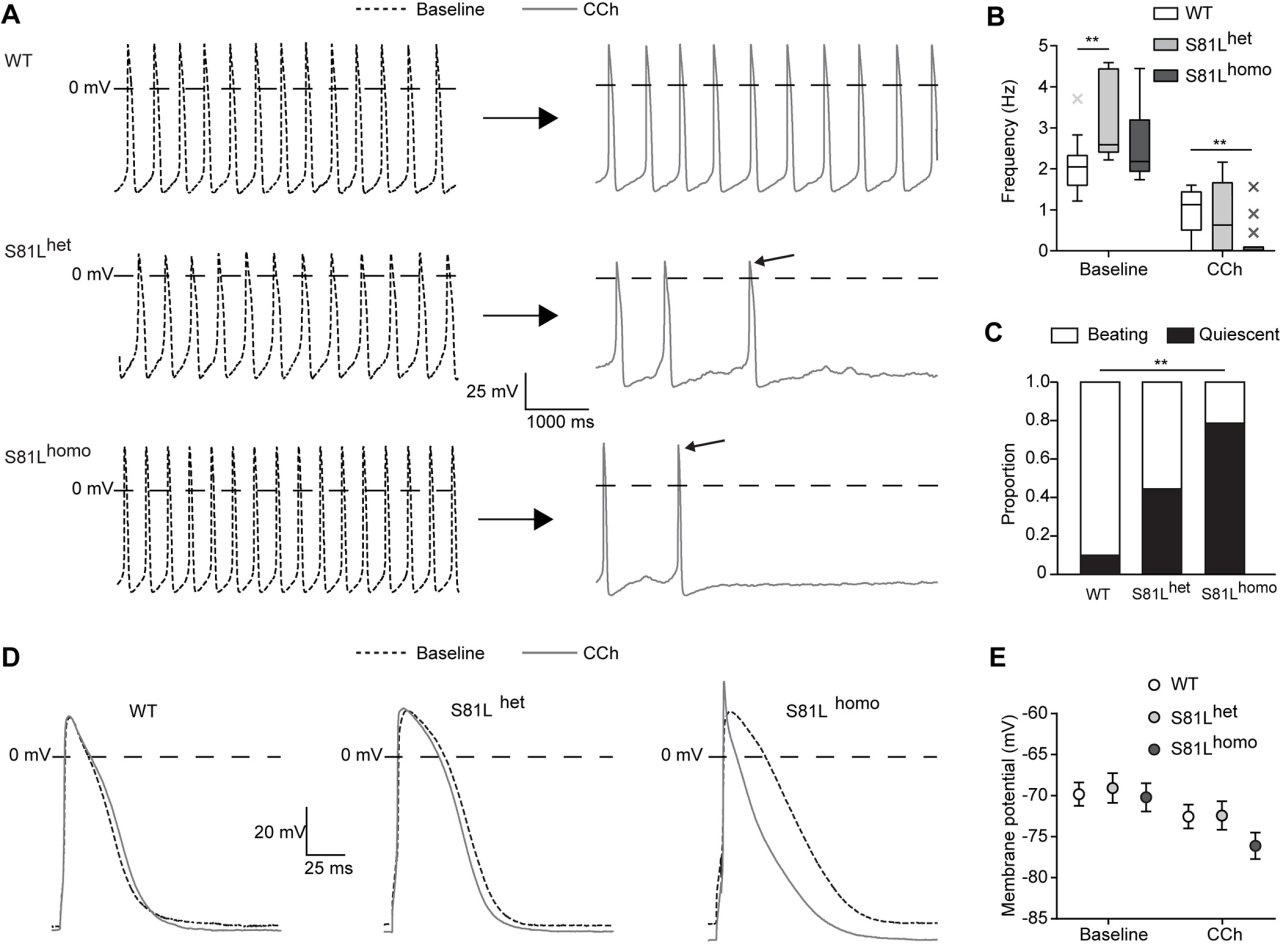
**CCh induces more pronounced effects in S81L^homo^ compared to WT and S81L^het^ RA-treated hiPSC-CMs.** (A) Representative spontaneous APs at baseline (left) and upon 10 µmol l^−1^ CCh (right) in WT, S81L^het^ and S81L^homo^ RA-treated hiPSC-CMs. The slanted arrows near the APs in the presence of CCh indicate the last AP before cessation of spontaneous activity. (B) Average frequencies before and during application of CCh. CCh drastically reduces the spontaneous AP frequency of S81L^homo^ hiPSC-CMs. WT, *n*=10; S81L^het^, *n*=9; S81L^homo^, *n*=14. **P<0.01 (two-way repeated measures ANOVA and post-hoc Bonferroni corrected pairwise comparison). (C) Proportion of WT, S81L^het^ and S81L^homo^ hiPSC-CMs that became quiescent upon application of CCh. WT, *n*=10; S81L^het^, *n*=9; S81L^homo^, *n*=14. **P<0.01 (chi-square test, followed by Bonferroni corrected pairwise comparisons). (D) Representative APs (1 Hz overdrive stimulation) before and after the application of CCh. (E) Average maximal diastolic potential (MDP) at baseline and upon CCh in WT, S81L^het^ and S81L^homo^ RA-treated hiPSC-CMs (1 Hz overdrive stimulation). WT, *n*=17; S81L^het^, *n*=10; S81L^homo^, *n*=13. Effect of CCh on MDP is significantly more pronounced in S81L^homo^ hiPSC-CMs (*P*<0.05 interaction effect CCh×genotype; two-way ANOVA).

Subsequently, we evaluated the effects of *I*_K,ACh_ activation on APs of WT, S81L^het^ and S81L^homo^ hiPSC-CMs. Figure 3A shows typical APs of spontaneously fast-beating hiPSC-CMs. Baseline AP parameters, i.e. in the absence of CCh, were similar between the three groups (Fig. 3A, and Table S4), with the exception of a slightly higher spontaneous beating frequency in S81L^het^ hiPSC-CMs (Fig. 3B), indicating that baseline electrophysiological properties are unaffected by the Gβ5 S81L variant. *I*_K,ACh_ activation by CCh induced a reduction in spontaneous beating frequency in all three hiPSC-CM lines, which was much more pronounced in S81L^homo^ compared to WT hiPSC-CMs (Fig. 3A-C). In fact, 79% of the S81L^homo^ hiPSC-CMs became quiescent, while a quiescent state was reached in 44 and 10% of S81L^het^ and WT hiPSC-CMs, respectively (Fig. 3C). This main finding in S81L^homo^ hiPSC-CMs was confirmed in a second separate S81L^homo^ clone. In this second clone, 71% of the spontaneously beating S81L^homo^ hiPSC-CMs (*n*=7) became quiescent upon application of CCh. The higher amount of spontaneous activity cessation in S81L^homo^ hiPSC-CMs is in agreement with the observed increased *I*_K,ACh_ density. Owing to the termination of the spontaneous activity, however, we were unable to determine the effects on AP characteristics. Therefore, we also assessed the effects of *I*_K,ACh_ activation on APs in slow-beating hiPSC-CMs stimulated at an overdrive stimulus frequency of 1 Hz. In the absence of CCh, AP parameters were not different between WT, S81L^het^ and S81L^homo^ hiPSC-CMs (Table 1). This further shows that the Gβ5-S81L variant does not affect electrophysiological properties at baseline conditions. Upon addition of CCh, a significant MDP hyperpolarization was observed in all three groups (Fig. 3D; Table 1). In S81L^homo^ hiPSC-CMs, however, hyperpolarization was more pronounced as compared to the other two groups (Fig. 3E; Table 1). Furthermore, S81L^homo^ hiPSC-CMs displayed a more pronounced shortening of AP duration at 20% (APD_20_; Table 1). This suggests that the AP configuration is only seriously affected by *I*_K,ACh_ in hiPSC-CMs if *I*_K,ACh_ densities are large. Thus, while AP parameters in the absence of CCh were largely identical in the three hiPSC-CMs lines, the S81L^homo^ line demonstrated a more pronounced effect of CCh in terms of cellular hyperpolarization, AP shortening and slowing of spontaneous beating, which is in line with both the increased *I*_K,ACh_ density and the bradycardia exhibited only by homozygous Gβ5-S81L patients (Lodder et al., 2016).

**Table 1.**
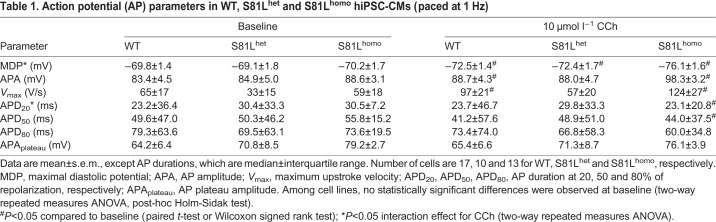
**Action potential (AP) parameters in WT, S81L^het^ and S81L^homo^ hiPSC-CMs (paced at 1 Hz)**

### Pharmacological *I*_K,ACh_ blockade rescues bradycardia in S81L^homo^ hiPSC-CMs and *gnb5-*knockout zebrafish

Given the observed increased *I*_K,ACh_ density in S81L^homo^ hiPSC-CMs, selective blockers of I(KACh) may provide a useful pharmacological treatment for patients with Gβ5 genetic defects. We tested this hypothesis in our hiPSC-CMs as well as *in vivo* in *gnb5*-knockout zebrafish that we generated previously (Lodder et al., 2016).

#### *I*_K,ACh_ blockade in hiPSC-CMs

**Fig. 4.**
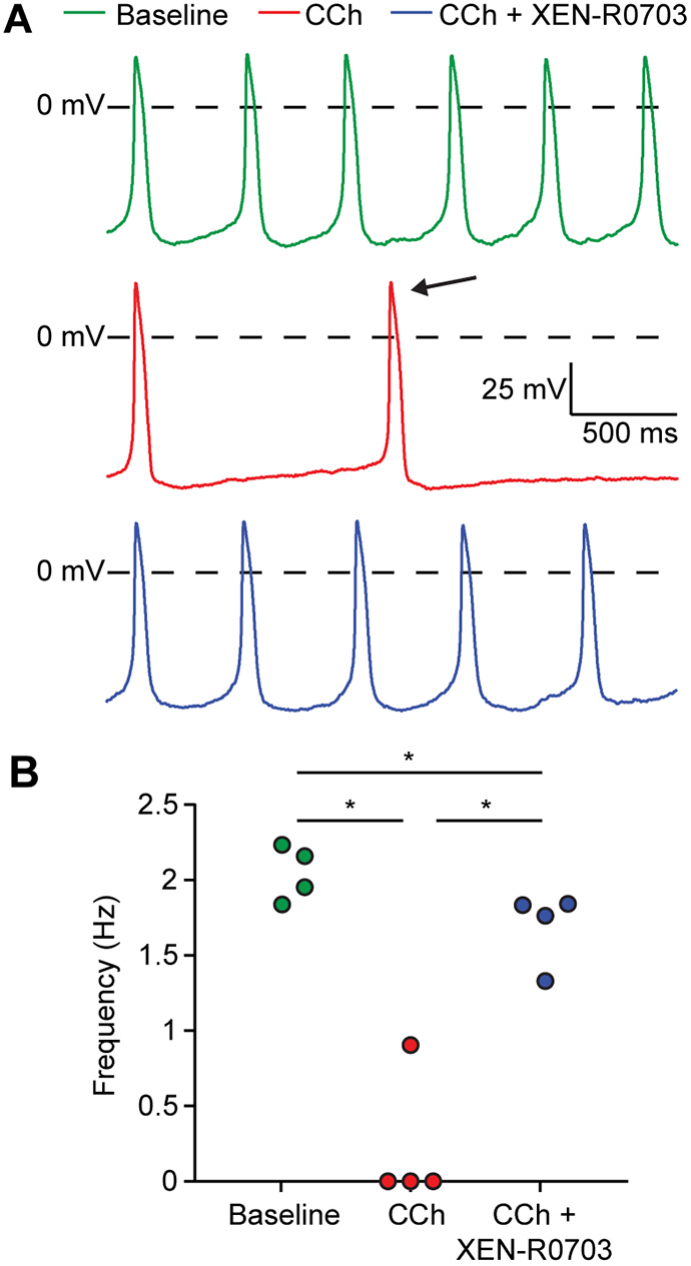
**CCh induces an extreme slowing of spontaneous beating in S81L^homo^ hiPSC-CMs, which is largely suppressed by *I*_K,ACh_ blockade through XEN-R0703.** (A) Typical APs of an S81L^homo^ hiPSC-CM at baseline (top), upon application of 10 µmol l^−1^ CCh (middle), and upon addition of 1 µmol l^−1^ XEN-R0703 in the presence of 10 µmol l^−1^ CCh (bottom). (B) Effects of CCh and XEN-R0703 on spontaneous AP frequency in four S81L^homo^ hiPSC-CMs. *P<0.05 (one-way repeated measures ANOVA followed by post-hoc Holm-Sidak test). XEN-R0703 restores the beating frequency of S81L^homo^ hiPSC-CMs to values similar to a baseline condition.

We evaluated possible pharmacological rescue of the phenotype in S81L^homo^ hiPSC-CMs using the selective I(KACh) blocker XEN-R0703 (Devalla et al., 2015). Figure 4A shows typical examples of spontaneous activity at baseline (top panel), in the presence of CCh (middle panel) and in the presence of CCh and XEN-R0703 (bottom panel). Addition of 1 µmol l^−1^ XEN-R0703 in the presence of 10 µmol l^−1^ CCh resulted in an almost complete reversal of the effects of CCh on spontaneous activity. This indicates that the drug is efficient in reversing the enhanced reduction of automaticity in S81L^homo^ hiPSC-CMs (Fig. 4B).

#### *I*_K,ACh_ blockade in zebrafish

**Fig. 5.**
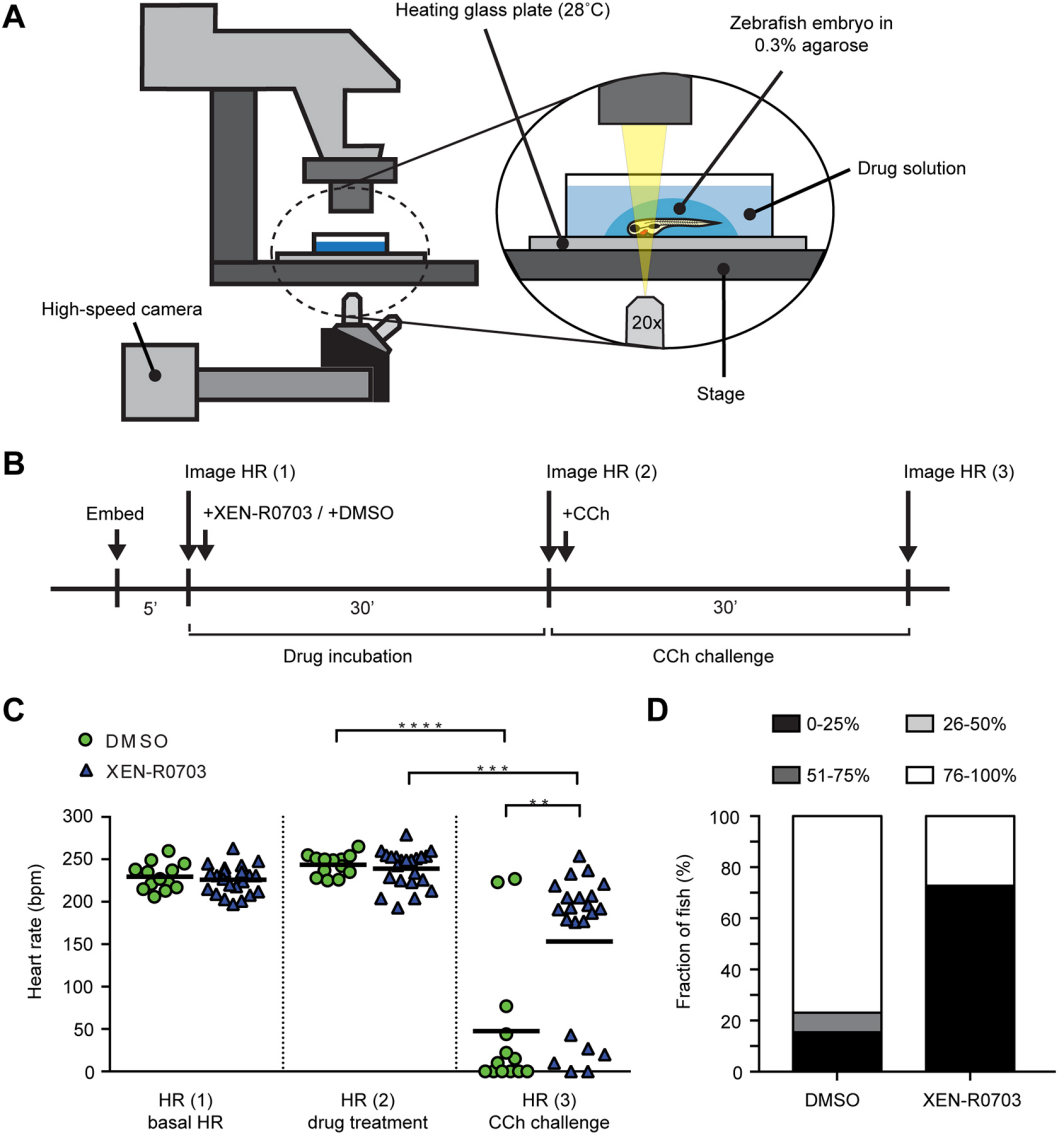
**Effect of *I*_K,ACh_ blockade on heart rate (HR) in *gnb5*-knockout zebrafish.** (A) Experimental setup for high-speed imaging of the zebrafish heart. HR was recorded at 150 fps for 10 s. (B) Time schedule of drug treatment and CCh challenge in *gnb5-*knockout larvae. (C) Scatter dot plots with mean of HR of *gnb5-*knockout larvae 5 days post-fertilization at basal level, after treatment with 50 µmol l^−1^ XEN-R0703 or DMSO, and upon application of 500 µmol l^−1^ CCh. DMSO, *n*=13; XEN-R0703, *n*=22. **P<0.01, ***P<0.001, ****P<0.0001 (two-way repeated measures ANOVA followed by Bonferroni corrected pairwise comparisons). (D) Percentage of *gnb5-*knockout larvae that responded to 500 µmol l^−1^ CCh with a decrease in HR of 0-25, 26-50, 51-75 or 76-100%. DMSO, *n*=13; XEN-R0703, *n*=22. Only 27% of *gnb5-*knockout larvae pretreated with XEN-R0703 displayed a pathological decrease in HR (51-75%, 76-100%), while over 80% of the not-pretreated *gnb5*-knockout larvae display the same decreased level of HR.

We also tested XEN-R0703 *in vivo* in a *gnb5*-knockout zebrafish model that faithfully recapitulates the phenotypical spectrum of patients with pathogenic variants in *GNB5*, including bradycardia (for details, see Lodder et al., 2016). In short, CCh led to a strong decrease in HR in *gnb5*-knockout zebrafish, while little effect was observed in wild-type and sibling larvae (Lodder et al., 2016). To study the effect of XEN-R0703, HR of *gnb5*-knockout zebrafish was first recorded at baseline, then after the administration of 50 µmol l^−1^ XEN-R0703 or DMSO, and lastly after challenging larvae with 500 µmol l^−1^ CCh (Fig. 5B). The used concentrations are higher than in our patch-clamp assay because the zebrafish assay is considerably less sensitive (Barros et al., 2008). While most larvae in the DMSO control group responded to CCh with a severe decrease in HR, larvae that were pretreated with XEN-R0703 had a markedly lower sensitivity to CCh (Fig. 5C). The response to CCh was calculated for each individual fish and the outcomes subdivided into categories of 0-25%, 26-50%, 51-75% and 76-100% decrease in HR. A 0-25% decrease in HR was considered physiological, whereas 26-100% was considered pathological. While 85% of the DMSO-treated *gnb5*-knockout fish showed a pathological decrease in HR, only 27% of fish pretreated with XEN-R0703 showed such a reduction (Fig. 5D). Together, these results demonstrate that *I*_K,ACh_ blockade largely rescues CCh-induced bradycardia in *gnb5*-knockout zebrafish.

### Predicted effects of the homozygous Gβ5-S81L variant in computational models of a SAN and an atrial cell

**Fig. 6.**
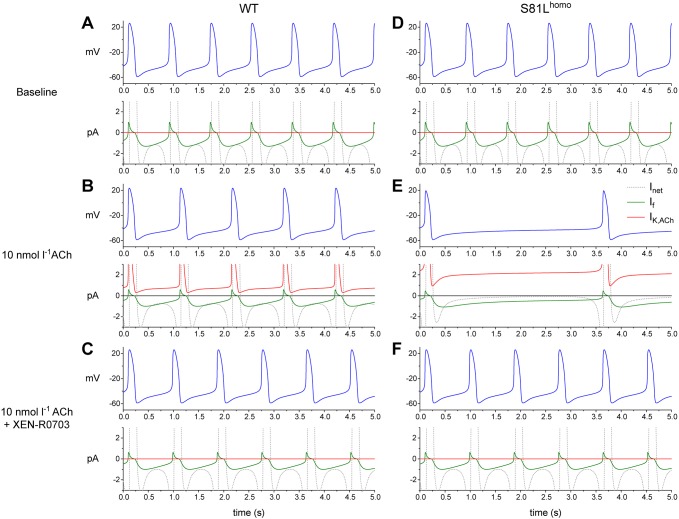
**ACh induces more pronounced effects in S81L^homo^ compared to WT** **human SAN pacemaker cells in computer simulations.** (A-C) Spontaneous APs (blue lines) and associated net membrane current (*I*_net_, gray lines), hyperpolarization-activated ‘funny current’ (*I*_f_, green lines) and *I*_K,ACh_ (red lines) in a WT human SAN pacemaker cell at baseline (A), upon addition of 10 nmol l^−1^ ACh (B) and upon addition of 10 nmol l^−1^ ACh in the presence of XEN-R0703 (C). (D-F) Spontaneous APs and associated *I*_net_, *I*_f_ and *I*_K,ACh_ in an S81L^homo^ human SAN pacemaker cell at baseline (D), upon addition of 10 nmol l^−1^ ACh (E) and upon addition of 10 nmol l^−1^ ACh in the presence of XEN-R0703 (F).

To evaluate whether the observed increase in *I*_K,ACh_ density, associated with the homozygous variant, is the major contributor to the generation of excessive bradycardia, we conducted computer simulations using mathematical models of both a SAN pacemaker cell and an atrial cell (Fabbri et al., 2017; Maleckar et al., 2009). We implemented a threefold increase in *I*_K,ACh_ density, as observed in our experiments in S81L^homo^ hiPSC-CMs (Fig. 2B). In the WT human SAN model, application of ACh at a concentration of 10 nmol l^−1^ activated *I*_K,ACh_ and inhibited the hyperpolarization-activated current (*I*_f_), resulting in a decrease in depolarizing net current (*I*_net_) during diastole. Consequently, beating rate decreased from 74 to 58 bpm (Fig. 6A,B). However, implementing a threefold-increased *I*_K,ACh_ density, which simulates the homozygous Gβ5-S81L variant effects, resulted in a dramatic slowing of beating rate (from 74 to 17 bpm) (Fig. 6D,E). Additionally, *I*_K,ACh_ blockade, which simulates the pharmacological effects of XEN-R0703, largely, but incompletely, reversed the effects of ACh (Fig. 6C,F). These findings closely resemble those obtained in S81L^homo^ hiPSC-CMs, demonstrating that the threefold increase in *I*_K,ACh_ density is sufficient to induce the bradycardic phenotype. The reversal of the ACh effects by the simulated application of XEN-R0703 is incomplete because the inhibiting effect of ACh on *I*_f_ persists. The inhibiting effect of ACh/CCh on *I*_f_ may also explain the incomplete rescue of beating frequency by XEN-0703 in the mutant zebrafish and hiPSC-CMs.

**Fig. 7.**
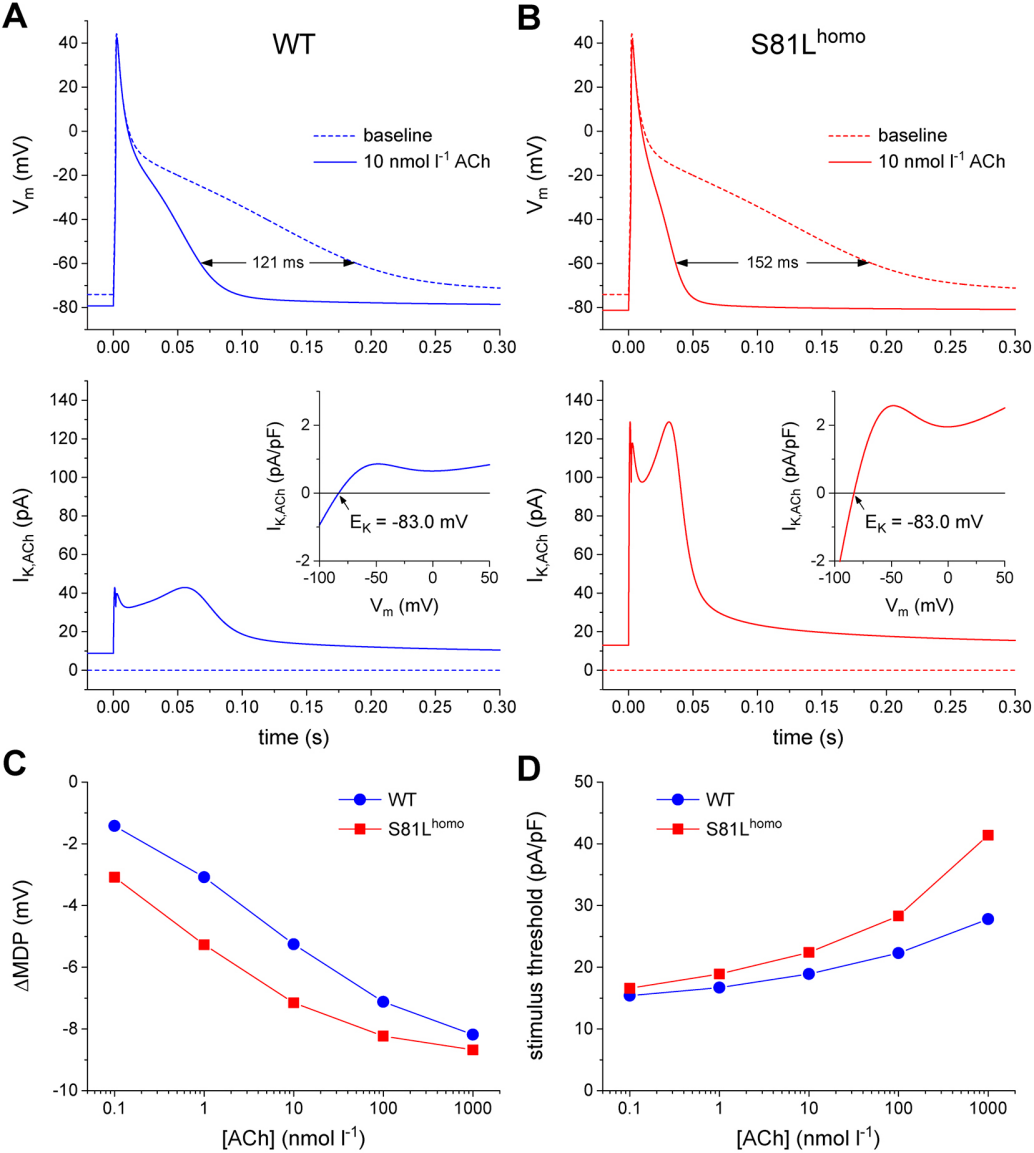
**ACh induces more pronounced AP effects in S81L^homo^ compared to WT human atrial myocytes in computer simulations.** (A,B) APs elicited at 1 Hz (top panels) and associated *I*_K,ACh_ (bottom panels) at baseline (dotted lines) and upon addition of 10 nmol l^−1^ ACh (solid lines) in WT (A) and S81L^homo^ (B) human atrial myocytes. The horizontal double-headed arrow in panel A indicates the ACh-induced shortening of AP duration. The ‘wobbles’ in the time course of *I*_K,ACh_ in panel B are caused by the N-shape of the *I*_K,ACh_ current-voltage relationship (insets). The slanted arrows in the insets point to the *I*_K,ACh_ reversal potential (*E*_K_) of −83.0 mV. (C,D) Shift in maximum diastolic potential (ΔMDP; C) and threshold stimulus current amplitude (D) at ACh concentrations ranging from 0.1 nmol l^−1^ to 1 µmol l^−1^. Note the logarithmic abscissa scale.

One may argue that the Gβ5-S81L variant further suppresses pacemaker activity by reducing the excitability of the atrial tissue surrounding the SAN through a more pronounced membrane hyperpolarization by CCh/ACh, as we observed in S81L^homo^ hiPSC-CMs (Fig. 3E). This was assessed in simulations of a human atrial CM. At baseline, the wild-type model cell exhibits an MDP of −74.1 mV, which becomes hyperpolarized by 5.3 mV upon application of 10 nmol l^−1^ ACh (Fig. 7A, top, and C) as a result of the activation of *I*_K,ACh_ (Fig. 7A, bottom). Owing to the hyperpolarization, the threshold-stimulus current amplitude increases by 29% (from 14.6 pA/pF at baseline to 18.9 pA/pF) (Fig. 7D). After implementing the threefold increase in *I*_K,ACh_ density (Fig. 7B), the ACh-induced hyperpolarization is larger (7.2 mV), as is the increase in threshold-stimulus current (53%). Qualitatively similar observations are made at other concentrations of ACh (Fig. 7C,D). Thus, the homozygous Gβ5-S81L variant likely does not only slow pacemaker activity of SAN cells, but also reduces excitability of atrial cells, and, as such, contributes to the bradycardic phenotype in patients.

## DISCUSSION

Genetic variants in *GNB5*, encoding Gβ5, have been linked to an autosomal recessive multisystem disorder that includes severe bradycardia at rest. The mechanism by which inherited genetic variation in *GNB5* causes bradycardia has remained unexplored. We here conducted electrophysiological studies in hiPSC-CMs that were genome-edited for the Gβ5-S81L variant to uncover the cellular mechanism underlying the bradycardia. We demonstrated that this variant causes a more pronounced slowing of beating rate by means of an increase in *I*_K,ACh_ density in response to cholinergic stimulation. Furthermore, we showed that this effect in hiPSC-CMs, as well as the effect of cholinergic-induced bradycardia in *gnb5*-knockout zebrafish, can be rescued by the specific *I*_K,ACh_ blocker XEN-R0703.

The cholinergic-gated K^+^ channel I(KACh) is involved in the negative chronotropic effect of the parasympathetic nervous system on HR (Wickman et al., 1998). It is an inwardly rectifying K^+^ channel (Sakmann et al., 1983), and its activation results in an outward current leading to membrane hyperpolarization, AP shortening and slowing of beating rate (DiFrancesco et al., 1989). The increased *I*_K,ACh_ density that we observed in S81L^homo^ hiPSC-CMs in response to cholinergic stimulation is thus a plausible explanation for the more pronounced slowing of beating rate and hyperpolarization we observed in these cells. Besides SAN cells, I(KACh) is also expressed in atrial CMs (Dobrzynski et al., 2001), which raises the possibility that the increase in *I*_K,ACh_ as a consequence of Gβ5-S81L homozygosity may also contribute to bradycardia during increased vagal tone by reducing the excitability of the atrium. Decreased atrial excitability favors SAN exit block due to source-to-sink mismatch, eventually resulting in SAN pauses and bradycardia (Joyner and van Capelle, 1986). Indeed, the computer simulation studies that we conducted in an atrial CM model revealed a decreased excitability in cells in which the experimentally observed increase in *I*_K,ACh_ was implemented. Thus, apart from a direct effect on intrinsic pacemaker function, a more pronounced atrial hyperpolarization due to increased *I*_K,ACh_ may also contribute to the phenotype of homozygous Gβ5-S81L variant carriers. The fact that the defect associated with the Gβ5-S81L variant in hiPSC-CMs became apparent only upon cholinergic stimulation is in line with clinical findings. Patients homozygous for this variant suffer from a severe reduction of minimum HR (dependent on the parasympathetic tone), while maximum HR (chronotropic competence) is normal (Lodder et al., 2016). Furthermore, the finding that S81L^het^ hiPSC-CMs exhibit a similar *I*_K,ACh_ density and a similar response to CCh compared to WT hiPSC-CMs is in line with the unaffected heterozygous carriers (Lodder et al., 2016).

The binding of ACh to M_2_ muscarinic receptors triggers the activation of heterotrimeric G-proteins that dissociate into Gα-GTP and Gβγ subunits. While Gβ1-β4/γ complexes function to activate GIRK channels, Gβ5 has an inhibitory effect on these channels (Lei et al., 2003). Gβ5 associates with RGS proteins of the R7 family, with RGS6 being the most abundant RGS in CMs. Gβ5-RGS complexes promote the hydrolysis of Gα-GTP to Gα-GDP, which then reassociates with Gβ1-β4/γ, likely relieving the activatory effect of Gβ1-β4/γ on the GIRK channel (Slepak, 2009). In a heterologous model of neuronal dopamine-receptor-mediated signaling, Shamseldin and colleagues showed that Gβ5-S81L leads to loss of function of Gβ5-RGS complexes [acting as GTPase-activating proteins (GAPs) on Gα-GTP], which presumably decreases the inhibitory reassociation of Gα-GDP with the βγ complexes during the termination response (Shamseldin et al., 2016). This would be in agreement with our observed increase in *I*_K,ACh_ density in the S81L^homo^ hiPSC-CMs. Conversely, a decreased rate of *I*_K,ACh_ deactivation was previously reported in both *Rgs6^−/−^* and *Gnb5^−/−^* mice (Posokhova et al., 2010; Wydeven et al., 2014; Yang et al., 2010). However, the mechanism by which decreased deactivation rate of *I*_K,ACh_ affects HR in the absence of changes in density remains unclear. In the present study, we did not find kinetic changes in *I*_K,ACh_ as a consequence of the Gβ5-S81L variant. Notwithstanding, the augmented *I*_K,ACh_ that we observe is in line with the HR-reducing effect of the variant in patients with the mutation, as substantiated by our computer simulations.

*I*_K,ACh_ augmentation as a disease mechanism is also operative during atrial fibrillation (Nattel et al., 2008). *I*_K,ACh_ blockers have been developed to treat atrial fibrillation, although so far with disappointing results (Podd et al., 2016). We demonstrated the potential of the *I*_K,ACh_ blocker XEN-R0703 by showing an almost complete rescue of the CCh-triggered decrease in beating frequency in S81L^homo^ hiPSC-CMs and by demonstrating rescue of CCh-induced bradycardia *in vivo* in *gnb5*-knockout zebrafish. Therefore, as previously suggested in mice (Mesirca et al., 2016), our current study provides a possible new application for *I*_K,ACh_ blockers as a therapy for bradycardia in patients carrying mutations in *GNB5*, which may substitute or postpone pacemaker treatment.

hiPSC-CMs have become increasingly used to study ion channel variants causing inherited arrhythmia syndromes (Bellin et al., 2012; Hoekstra et al., 2012). Yet, differentiation protocols that have been used thus far in disease modeling generate primarily ventricular-like CMs (Devalla et al., 2015). We here investigated for the first time the effects of a human genetic variant in a signal transduction component utilizing a specific differentiation protocol that enriches for hiPSC-CMs expressing I(KACh), a channel that is active in SAN and atrial cells. This cellular model enabled us to study the effect of genetic variation in a component of a G protein-coupled receptor signaling cascade that impacts on ion channel function, rather than genetic variation within the ion channel itself, further underscoring the potential of this model. The introduction of the genetic variant in both the heterozygous and the homozygous state allowed us to compare the effect of different states of the variant, which matched the phenotype observed in heterozygous and homozygous S81L^homo^ carriers.

While the effects of the mutation on *I*_K,ACh_ are in agreement with the bradycardia phenotype of the patients, we have not studied the effect of the Gβ5-S81L variant in ‘pacemaker-like’ cells derived from hiPSCs (Birket et al., 2015; Protze et al., 2017). Furthermore, we focused on the effects of the Gβ5-S81L variant on intrinsic CM electrophysiological properties. However, *GNB5* is also expressed in neurons. Therefore, we cannot exclude the possibility of an effect of the variant on the beating rate through its effect in central or peripheral neurons. Finally, baseline AP properties were not different between groups, suggesting that the Gβ5-S81L variant does not affect ion currents at baseline. However, we cannot exclude effects on other ion currents that are modulated by CCh that may contribute to the bradycardic phenotype as well. Nonetheless, our modeling study demonstrated that the effect of the variant on *I*_K,ACh_ density is the main contributor to the reduction of the beating rate at the CM level and that blockade of the channel almost completely reverses the bradycardic phenotype.

We studied for the first time the cellular electrophysiological consequences of human genetic variation in *GNB5*. We demonstrated that the Gβ5-S81L variant in the homozygous state results in an increased *I*_K,ACh_ density upon muscarinic receptor stimulation and excessive slowing of spontaneous activity, a plausible mechanism for the bradycardia observed in the patients. Pharmacological *I*_K,ACh_ blockade rescues the phenotype in a cellular and an *in vivo* model, pointing to a possible therapy for bradycardia in patients carrying pathogenic variants in *GNB5*.

## MATERIALS AND METHODS

### Genetic modification and differentiation of hiPSC lines into CMs

The study was approved by the Medical Ethics Committee of the Amsterdam UMC, location AMC (NL30225.018.09), and the University Medical Center Göttingen (Az 21/1/11). Written informed consent was obtained from the control individual from whom the hiPSC line was generated. The (*GNB5* c.242C>T) Gβ5-S81L variant was introduced in the heterozygous or homozygous state into a previously characterized control hiPSC line (Dudek et al., 2013) by means of CRISPR/Cas9-based genome editing, according to a previously published protocol (Ran et al., 2013). Briefly, we designed the *GNB5*-targeting gRNA sequence close to the single-nucleotide variant of interest (Fig. S1B and Table S3) and cloned it into the pSPCas9(BB)-2a-GFP vector. Next, 4 µg of the gRNA-expressing vector and 4 µg of a single-strand oligodeoxynucleotide (ssODN) carrying: (1) the c.242C>T mutation, (2) a total of 96 nucleotides of homologous sequence flanking the mutation site, and (3) a synonymous mutation that generates a *Hin*dIII restriction site (Fig. S1B), were delivered into the hiPSCs by nucleofection using the Lonza Amaxa 2b Nucleofector Device and the Stem Cell Nucleofector I Kit according to manufacturers’ protocols (Fig. S1A). Restriction analysis of a PCR fragment defined by the *GNB5*-screen primers (Table S1) was used to verify the presence of cells carrying the homologous recombination event in the whole population of transfected cells. GFP^+^ transfected cells that had internalized the gene-editing machinery were subsequently selected by fluorescent-activated cell sorting (FACS) and seeded at a low density (limiting dilution) to achieve monoclonal growth. A total of 96 separate clones were isolated mechanically, transferred into separate wells of a 96-well plate and expanded. Of these, 86 clones could be screened by Sanger sequencing for the presence of the *GNB5* mutation, a region of approximately 1000 nucleotides surrounding the gRNA target site. Nine clones were homozygous, two were heterozygous, 73 exhibited a mixed genetic profile [e.g. indels; these included clones that were heterozygous (one clone) and homozygous (12 clones) at the nucleotide of interest] and two clones contained a wild-type sequence. Heterozygous and homozygous clones carrying the desired S81L *GNB5* mutation were further expanded and cryo-stored. Clones with the desired genotype for further functional analysis were randomly picked from those available. Furthermore, using the CRISPR Finder online tool (Hodgkins et al., 2015), we identified three putative off-target sequences of the gRNA used (Table S3). Sanger sequencing of these regions (primers in Table S1) in the clones that were used in the functional studies did not reveal any sequence variant differences. Additionally, in order to exclude the possibility that a large deletion occurred on one of the two alleles at the targeted region (that, due to hemizygosity, could mimic the homozygous state on the DNA sequence read) we demonstrated that the heterozygous SNPs (rs28437132 and rs28409853, present in the original hiPSC line) were maintained within the same PCR product as the *GNB5* variant.

The original and generated hiPSC lines tested negative for mycoplasma contaminations. However, they were not recently authenticated by STR analysis. The generated isogenic hiPSC lines were subject to quality control including the evaluation of expression of the pluripotency markers TRA1-81, OCT-4, NANOG and SSEA4 by immunocytochemistry (Fig. S1D,E and Table S2) as described previously (Veerman et al., 2016). Karyotype analysis was performed by the COBRA Fish technique (Szuhai and Tanke, 2006) for the WT and S81L^het^ hiPSC line and by G-banding for the S81L^homo^ hiPSC line (Fig. S1F,G).

HiPSCs were differentiated into CMs that robustly express I(KACh) using a previously described protocol in chemically defined medium and serum-free and feeder-free conditions (Lian et al., 2013). One µmol l^−1^ all-trans-retinoic acid (RA; Sigma) was applied during day 4-7 of differentiation to promote acquisition of atrial-like fate and expression of the I(KACh) channel (Devalla et al., 2015; Marczenke et al., 2017). A metabolic selection-based enrichment for hiPSC-CMs was used by applying glucose-depleted culture medium containing 4 mmol l^−1^ lactate during day 20-26 of differentiation (Tohyama et al., 2013). For electrophysiological experiments, hiPSC-CM cultures were enzymatically dissociated into single cells and plated at a low density on Matrigel-coated coverslips (Meijer van Putten et al., 2015). All experiments were performed on cells from at least three independent differentiation replicas.

### RT-qPCR

RNA was isolated from the three isogenic hiPSC lines by using the Macherey-Nagel NucleoSpin RNA II isolation kit following the manufacturer's instructions. RNA (500 ng) was retro-transcribed using SuperScript II Reverse Transcriptase (Life Technologies) and oligo dT primers. To determine transcript abundance, quantitative PCR (qPCR) was conducted with SYBR Green on a Roche LightCycler 480 Real-Time PCR System. RNA samples were obtained from three biological differentiation replicas with triplicate measurements (technical replicas). Gene expression levels were analyzed using the LinReg PCR program (Ramakers et al., 2003). *TBP* (TATA binding protein) was used as a reference gene. Primers are listed in Table S1.

### Cellular electrophysiology in hiPSC-CMs

#### Data acquisition

*I*_K,ACh_ and APs were measured using an Axopatch 200B amplifier (Molecular Devices, Sunnyvale, CA, USA). Voltage control, data acquisition and analysis were realized with custom software. Pipettes (resistance 2-3 MΩ) were pulled from borosilicate glass capillaries (Harvard Apparatus, UK). Cell membrane capacitance (*C*_m_) was calculated and potentials were corrected for calculated liquid junction potentials (Barry and Lynch, 1991). Signals were low-pass-filtered with a cut-off of 5 kHz, and digitized at 40 and 20 kHz for APs and *I*_K,ACh_, respectively.

#### *I*_K,ACh_ measurements

*I*_K,ACh_ was measured at 21±0.2°C using the ruptured patch-clamp technique. Bath solution contained (in mmol l^−1^): 145 KCl, 1.0 MgCl_2_, 1.8 CaCl_2_, 10 glucose, 5 HEPES, pH 7.4 (KOH). Pipettes were filled with solution containing (in mmol l^−1^): 105 K-gluc, 20 KCl, 5 NaCl, 1 MgCl_2_, 10 BAPTA (pre-dissolved in KOH), 5 MgATP, 0.3 Na-GTP, 10 HEPES, pH 7.2 (NMDG-OH). *I*_K,ACh_ was elicited at −50 mV by rapidly applying 100 µmol l^−1^ CCh (Sigma) using a puffer pipette system in close proximity to the patched cell. *I*_K,ACh_ deactivation time kinetics were measured in the presence of 1 mmol l^−1^ of atropine, a muscarinic receptor antagonist. Current density was calculated by dividing the CCh-elicited current by *C*_m_. Time dependence of activation and deactivation were determined by fitting the mono-exponential functions *I*/*I*_max_=A×[1-exp(-t/τ)] and *I*/*I*_max_=A×exp(-t/τ), respectively, in which τ is the time constant of activation and deactivation, respectively; *I* and *I*_max_ are current (mA); *A* is amplitude; and *t* is time (seconds). Desensitization was defined as the percentage of current decay at steady state during continuous exposure to CCh. Cells that virtually lacked *I*_K,ACh_ were excluded from analysis. The proportion of excluded cells was similar in all groups.

#### AP measurements

APs were recorded at 36±0.2°C using the amphotericin-perforated patch-clamp technique and solutions as described previously (Devalla et al., 2015; Marczenke et al., 2017; Meijer van Putten et al., 2015; Verkerk et al., 2017). APs were elicited at an overdrive stimulus frequency of 1 Hz with 3-ms 1.2× threshold current pulses through the patch pipette. APs were recorded under control conditions and after 4-5 min of application of 10 µmol l^−1^ CCh. We analyzed cycle length, MDP, AP amplitude (APA), AP plateau amplitude (APA_plateau_; measured 20 ms after initiation of the AP upstroke), maximum AP upstroke velocity (*V*_max_), and AP duration at 20, 50 and 80% repolarization (APD_20_, APD_50_ and APD_80_, respectively). Parameters from ten consecutive APs were averaged.

#### hiPSC-CM selection for assessment of CCh effect on AP

Two populations of cells were included, one with a beating frequency >1 Hz and another population with a beating frequency <1 Hz. The first population was used to assess the effects of CCh on beating frequency (shown in Fig. 3A,B). Since a large proportion of cells became quiescent upon CCh application, the effects on AP properties could not be evaluated. Therefore, we also selected a population of cells that exhibited a beating rate <1 Hz, which were paced at a fixed rate of 1 Hz. The results of the latter population are shown in Fig. 3D,E and in Table 1.

### Heart rate analysis in *gnb5*-knockout zebrafish

Zebrafish experiments were conducted in accordance with the local ethical guidelines and approved by the Ethics Committee of the Royal Netherlands Academy of Arts and Sciences. Fishes were housed under standard conditions as previously described (Westerfield, 2000). Fish with a homozygous loss-of-function mutation in *gnb5a* and a heterozygous loss-of-function mutation in *gnb5b* (*gnb5a*^−/−^ *gnb5b*^+/−^) were crossed to obtain double-knockout mutants (*gnb5a*^−/−^ *gnb5b*^−/−^; referred to as *gnb5* knockout) as described previously (Lodder et al., 2016). Embryos at 5 days post-fertilization were embedded in 0.3% agarose (UltraPure, Thermo Fisher Scientific) prepared in E3 medium containing 16 mg ml^−1^ Tricaine. Measurements were performed at 28°C using an inverted light microscope with climate chamber and a high-speed CCD camera (Hamamatsu Photonics K.K., Hamamatsu City, Japan). HR was analyzed using ImageJ (National Institutes of Health, Bethesda, MD, USA).

### Computer simulations

The spontaneous electrical activity of a single human SA nodal pacemaker cell was simulated using the comprehensive mathematical model that was recently developed by Fabbri et al. (Fabbri et al., 2017). In this Fabbri-Severi model, ACh does not only activate the ACh-activated K^+^ current (*I*_K,ACh_), but also inhibits the hyperpolarization-activated ‘funny current’ (*I*_f_), through a hyperpolarizing shift in its voltage dependence of activation (thus shifting the half-activation voltage of *I*_f_ to a more negative value). Furthermore, ACh inhibits the L-type Ca^2+^ current (*I*_Ca,L_) and the sarco/endoplasmic reticulum Ca^2+^-ATPase (SERCA pump), which regulates Ca^2+^ uptake into the sarcoplasmic reticulum. Under control conditions, the simulated administration of 10 nmol l^−1^ ACh leads to a 21% reduction in pacemaking rate from 74 to 58 bpm. Of note, the inhibition of *I*_f_ and activation of *I*_K,ACh_ are the major determinants of this rate slowdown, whereas the inhibiting effects on *I*_Ca,L_ and the SERCA pump only play a minor role (Fabbri et al., 2017).

For simulations of a single human atrial cell, we used the model by Maleckar et al. (2009). The latter model, which is also known as the ‘human atrial myocyte with new repolarization’ (hAMr) model, was selected because it includes well-validated equations for *I*_K,ACh_ (Maleckar et al., 2008). APs were elicited with a 2-ms ≈50% suprathreshold stimulus current at a frequency of 1 Hz. The threshold-stimulus current amplitude was determined by increasing the stimulus current amplitude in 0.1-pA/pF steps until a train of 100 APs could successfully be elicited. To prevent slow drifts in ion concentrations, the intracellular Na^+^ and K^+^ concentrations were fixed, as were the cleft ion concentrations.

The CellML code of both models, as available from the CellML Model Repository (Lloyd et al., 2008), was edited and run in version 0.9.31.1409 of the Windows-based Cellular Open Resource (COR) environment (Garny et al., 2003). In both models, the S81L^homo^ mutation was simulated through a threefold increase in the conductance of I(KACh), based on the experimental observations shown in Fig. 2. All simulations were run for a sufficiently long time, i.e. for the duration of a train of 200 APs, to reach steady-state behavior.

### Statistical analyses

Statistical analysis was performed in IBM SPSS statistics 24 and GraphPad Prism 7. Data are presented as mean±s.e.m., unless stated otherwise. For non-normally distributed parameters, data are presented as boxplots, in which boxes represent lower quartile, median and upper quartile, and whiskers 1.5× interquartile range. Parameters were tested for normal distribution and equality of variance with the Shapiro-Wilk and Levene's test, respectively. In case of normally distributed data, two-sided *t*-tests or paired *t*-tests were performed for two groups and one-way ANOVA, followed by post-hoc Holm-Sidak tests for >2 groups. In case of a non-normal distribution, Mann–Whitney *U*-tests or Wilcoxon rank tests were used for two groups and Kruskal–Wallis tests, followed by Bonferroni corrected pairwise comparisons, for >2 groups. To test for differences in proportions, a chi-square test was used. In hiPSC-CMs, drug effects and effects of CCh were tested by one-way or two-way repeated measures ANOVA, respectively, followed by Holm-Sidak post-hoc tests or Bonferroni corrected pairwise comparisons, respectively. In zebrafish, the effect of XEN-R0703 was evaluated by two-way repeated measures ANOVA, followed by post-hoc Bonferroni corrected pairwise comparisons. *P*<0.05 defines statistical significance.

## Supplementary Material

Supplementary information

## References

[DMM037994C1] Barros T. P., Alderton W. K., Reynolds H. M., Roach A. G. and Berghmans S. (2008). Zebrafish: an emerging technology for in vivo pharmacological assessment to identify potential safety liabilities in early drug discovery. *Br. J. Pharmacol.* 154, 1400-1413. 10.1038/bjp.2008.249 18552866 PMC2492106

[DMM037994C2] Barry P. H. and Lynch J. W. (1991). Liquid junction potentials and small cell effects in patch-clamp analysis. *J. Membr. Biol.* 121, 101-117. 10.1007/BF01870526 1715403

[DMM037994C3] Bellin M., Marchetto M. C., Gage F. H. and Mummery C. L. (2012). Induced pluripotent stem cells: the new patient? *Nat. Rev. Mol. Cell Biol.* 13, 713-726. 10.1038/nrm3448 23034453

[DMM037994C4] Birket M. J., Ribeiro M. C., Verkerk A. O., Ward D., Leitoguinho A. R., den Hartogh S. C., Orlova V. V., Devalla H. D., Schwach V., Bellin M. et al. (2015). Expansion and patterning of cardiovascular progenitors derived from human pluripotent stem cells. *Nat. Biotechnol.* 33, 970-979. 10.1038/nbt.3271 26192318

[DMM037994C5] Devalla H. D., Schwach V., Ford J. W., Milnes J. T., El-Haou S., Jackson C., Gkatzis K., Elliott D. A., Chuva de Sousa Lopes S. M., Mummery C. L. et al. (2015). Atrial-like cardiomyocytes from human pluripotent stem cells are a robust preclinical model for assessing atrial-selective pharmacology. *EMBO Mol. Med.* 7, 394-410. 10.15252/emmm.201404757 25700171 PMC4403042

[DMM037994C6] DiFrancesco D., Ducouret P. and Robinson R. B. (1989). Muscarinic modulation of cardiac rate at low acetylcholine concentrations. *Science* 243, 669-671. 10.1126/science.2916119 2916119

[DMM037994C7] Dobrzynski H., Marples D. D. R., Musa H., Yamanushi T. T., Henderson Z., Takagishi Y., Honjo H., Kodama I. and Boyett M. R. (2001). Distribution of the muscarinic K^+^ channel proteins Kir_3.1_ and Kir_3.4_ in the ventricle, atrium, and sinoatrial node of heart. *J. Histochem. Cytochem.* 49, 1221-1234. 10.1177/002215540104901004 11561006

[DMM037994C8] Dudek J., Cheng I.-F., Balleininger M., Vaz F. M., Streckfuss-Bömeke K., Hübscher D., Vukotic M., Wanders R. J. A., Rehling P. and Guan K. (2013). Cardiolipin deficiency affects respiratory chain function and organization in an induced pluripotent stem cell model of Barth syndrome. *Stem Cell Res.* 11, 806-819. 10.1016/j.scr.2013.05.005 23792436

[DMM037994C9] Fabbri A., Fantini M., Wilders R. and Severi S. (2017). Computational analysis of the human sinus node action potential: model development and effects of mutations. *J. Physiol.* 595, 2365-2396. 10.1113/JP273259 28185290 PMC5374121

[DMM037994C10] Garny A., Kohl P. and Noble D. (2003). Cellular open resource (COR): a public CellML based environment for modelling biological function. *Int. J. Bifurcat. Chaos.* 13, 3579-3590. 10.1142/S021812740300882X

[DMM037994C11] Hodgkins A., Farne A., Perera S., Grego T., Parry-Smith D. J., Skarnes W. C. and Iyer V. (2015). WGE: a CRISPR database for genome engineering. *Bioinformatics* 31, 3078-3080. 10.1093/bioinformatics/btv308 25979474 PMC4565030

[DMM037994C12] Hoekstra M., Mummery C. L., Wilde A. A. M., Bezzina C. R. and Verkerk A. O. (2012). Induced pluripotent stem cell derived cardiomyocytes as models for cardiac arrhythmias. *Front. Physiol.* 3, 346 10.3389/fphys.2012.00346 23015789 PMC3449331

[DMM037994C13] Joyner R. W. and van Capelle F. J. (1986). Propagation through electrically coupled cells. How a small SA node drives a large atrium. *Biophys. J.* 50, 1157-1164. 10.1016/S0006-3495(86)83559-7 3801575 PMC1329789

[DMM037994C14] Kim J. J., Yang L., Lin B., Zhu X., Sun B., Kaplan A. D., Bett G. C. L., Rasmusson R. L., London B. and Salama G. (2015). Mechanism of automaticity in cardiomyocytes derived from human induced pluripotent stem cells. *J. Mol. Cell. Cardiol.* 81, 81-93. 10.1016/j.yjmcc.2015.01.013 25644533 PMC4409767

[DMM037994C15] Krapivinsky G., Gordon E. A., Wickman K., Velimirovic B., Krapivinsky L. and Clapham D. E. (1995). The G-protein-gated atrial K^+^ channel I_KACh_ is a heteromultimer of two inwardly rectifying K^+^-channel proteins. *Nature* 374, 135-141. 10.1038/374135a0 7877685

[DMM037994C16] Lei Q., Jones M. B., Talley E. M., Garrison J. C. and Bayliss D. A. (2003). Molecular mechanisms mediating inhibition of G protein-coupled inwardly-rectifying K+ channels. *Mol. Cells* 15, 1-9. 12661754

[DMM037994C17] Lek M., Karczewski K. J., Minikel E. V., Samocha K. E., Banks E., Fennell T., O'Donnell-Luria A. H., Ware J. S., Hill A. J., Cummings B. B. et al. (2016). Analysis of protein-coding genetic variation in 60,706 humans. *Nature* 536, 285-291. 10.1038/nature19057 27535533 PMC5018207

[DMM037994C18] Lian X., Zhang J., Azarin S. M., Zhu K., Hazeltine L. B., Bao X., Hsiao C., Kamp T. J. and Palecek S. P. (2013). Directed cardiomyocyte differentiation from human pluripotent stem cells by modulating Wnt/beta-catenin signaling under fully defined conditions. *Nat. Protoc.* 8, 162-175. 10.1038/nprot.2012.150 23257984 PMC3612968

[DMM037994C19] Lloyd C. M., Lawson J. R., Hunter P. J. and Nielsen P. F. (2008). The CellML model repository. *Bioinformatics* 24, 2122-2123. 10.1093/bioinformatics/btn390 18658182

[DMM037994C20] Lodder E. M., De Nittis P., Koopman C. D., Wiszniewski W., Moura de Souza C. F., Lahrouchi N., Guex N., Napolioni V., Tessadori F., Beekman L. et al. (2016). GNB5 Mutations cause an autosomal-recessive multisystem syndrome with sinus bradycardia and cognitive disability. *Am. J. Hum. Genet.* 99, 704-710. 10.1016/j.ajhg.2016.06.025 27523599 PMC5010642

[DMM037994C21] Ma J., Guo L., Fiene S. J., Anson B. D., Thomson J. A., Kamp T. J., Kolaja K. L., Swanson B. J. and January C. T. (2011). High purity human-induced pluripotent stem cell-derived cardiomyocytes: electrophysiological properties of action potentials and ionic currents. *Am. J. Physiol. Heart Circ. Physiol.* 301, H2006-H2017. 10.1152/ajpheart.00694.2011 21890694 PMC4116414

[DMM037994C22] Maleckar M. M., Greenstein J. L., Trayanova N. A. and Giles W. R. (2008). Mathematical simulations of ligand-gated and cell-type specific effects on the action potential of human atrium. *Prog. Biophys. Mol. Biol.* 98, 161-170. 10.1016/j.pbiomolbio.2009.01.010 19186188 PMC2836896

[DMM037994C23] Maleckar M. M., Greenstein J. L., Giles W. R. and Trayanova N. A. (2009). K+ current changes account for the rate dependence of the action potential in the human atrial myocyte. *Am. J. Physiol. Heart Circ. Physiol.* 297, H1398-H1410. 10.1152/ajpheart.00411.2009 19633207 PMC2770776

[DMM037994C24] Marczenke M., Piccini I., Mengarelli I., Fell J., Röpke A., Seebohm G., Verkerk A. O. and Greber B. (2017). Cardiac subtype-specific modeling of Kv1.5 ion channel deficiency using human pluripotent stem cells. *Front. Physiol.* 8, 469 10.3389/fphys.2017.00469 28729840 PMC5498524

[DMM037994C25] Meijer van Putten R. M., Mengarelli I., Guan K., Zegers J. G., van Ginneken A. C., Verkerk A. O. and Wilders R. (2015). Ion channelopathies in human induced pluripotent stem cell derived cardiomyocytes: a dynamic clamp study with virtual I_K1_. *Front. Physiol.* 6, 7 10.3389/fphys.2015.00007 25691870 PMC4315032

[DMM037994C26] Mesirca P., Bidaud I., Briec F., Evain S., Torrente A. G., Le Quang K., Leoni A.-L., Baudot M., Marger L., Chung You Chong A. et al. (2016). G protein-gated I_KACh_ channels as therapeutic targets for treatment of sick sinus syndrome and heart block. *Proc. Natl. Acad. Sci. USA* 113, E932-E941. 10.1073/pnas.1517181113 26831068 PMC4763776

[DMM037994C27] Nattel S., Burstein B. and Dobrev D. (2008). Atrial remodeling and atrial fibrillation: mechanisms and implications. *Circ. Arrhythm Electrophysiol.* 1, 62-73. 10.1161/CIRCEP.107.754564 19808395

[DMM037994C28] Podd S. J., Freemantle N., Furniss S. S. and Sulke N. (2016). First clinical trial of specific I_KACh_ blocker shows no reduction in atrial fibrillation burden in patients with paroxysmal atrial fibrillation: pacemaker assessment of BMS 914392 in patients with paroxysmal atrial fibrillation. *Europace* 18, 340-346. 10.1093/europace/euv263 26462707

[DMM037994C29] Posokhova E., Wydeven N., Allen K. L., Wickman K. and Martemyanov K. A. (2010). RGS6/Gbeta5 complex accelerates I_KACh_ gating kinetics in atrial myocytes and modulates parasympathetic regulation of heart rate. *Circ. Res.* 107, 1350-1354. 10.1161/CIRCRESAHA.110.224212 20884879 PMC3014848

[DMM037994C30] Protze S. I., Liu J., Nussinovitch U., Ohana L., Backx P. H., Gepstein L. and Keller G. M. (2017). Sinoatrial node cardiomyocytes derived from human pluripotent cells function as a biological pacemaker. *Nat. Biotechnol.* 35, 56-68. 10.1038/nbt.3745 27941801

[DMM037994C31] Ramakers C., Ruijter J. M., Deprez R. H. and Moorman A. F. (2003). Assumption-free analysis of quantitative real-time polymerase chain reaction (PCR) data. *Neurosci. Lett.* 339, 62-66. 10.1016/S0304-3940(02)01423-4 12618301

[DMM037994C32] Ran F. A., Hsu P. D., Wright J., Agarwala V., Scott D. A. and Zhang F. (2013). Genome engineering using the CRISPR-Cas9 system. *Nat. Protoc.* 8, 2281-2308. 10.1038/nprot.2013.143 24157548 PMC3969860

[DMM037994C33] Sakmann B., Noma A. and Trautwein W. (1983). Acetylcholine activation of single muscarinic K+ channels in isolated pacemaker cells of the mammalian heart. *Nature* 303, 250-253. 10.1038/303250a0 6302520

[DMM037994C34] Shamseldin H. E., Masuho I., Alenizi A., Alyamani S., Patil D. N., Ibrahim N., Martemyanov K. A. and Alkuraya F. S. (2016). *GNB5* mutation causes a novel neuropsychiatric disorder featuring attention deficit hyperactivity disorder, severely impaired language development and normal cognition. *Genome Biol.* 17, 195 10.1186/s13059-016-1061-6 27677260 PMC5037613

[DMM037994C35] Slepak V. Z. (2009). Structure, function, and localization of Gbeta5-RGS complexes. *Prog. Mol. Biol. Transl. Sci.* 86, 157-203. 10.1016/S1877-1173(09)86006-7 20374716 PMC3312022

[DMM037994C50] Stallmeyer B., Kuss J., Kotthoff S., Zumhagen S., Vowinkel K. S., Rinne S., Matschke L. A., Friedrich C., Schulze-Bahr E., Rust S. et al (2017). A mutation in the G-protein gene GNB2 causes familial sinus node and atrioventricular conduction dysfunction. *Circ. Res.* 120, e33-e44. 10.1161/circresaha.116.310112 28219978

[DMM037994C36] Szuhai K. and Tanke H. J. (2006). COBRA: combined binary ratio labeling of nucleic-acid probes for multi-color fluorescence in situ hybridization karyotyping. *Nat. Protoc.* 1, 264-275. 10.1038/nprot.2006.41 17406243

[DMM037994C37] Tohyama S., Hattori F., Sano M., Hishiki T., Nagahata Y., Matsuura T., Hashimoto H., Suzuki T., Yamashita H., Satoh Y. et al. (2013). Distinct metabolic flow enables large-scale purification of mouse and human pluripotent stem cell-derived cardiomyocytes. *Cell Stem Cell* 12, 127-137. 10.1016/j.stem.2012.09.013 23168164

[DMM037994C38] Turkdogan D., Usluer S., Akalin F., Agyuz U. and Aslan E. S. (2017). Familial early infantile epileptic encephalopathy and cardiac conduction disorder: A rare cause of SUDEP in infancy. *Seizure* 50, 171-172. 10.1016/j.seizure.2017.06.019 28697420

[DMM037994C39] Veerman C. C., Kosmidis G., Mummery C. L., Casini S., Verkerk A. O. and Bellin M. (2015). Immaturity of human stem-cell-derived cardiomyocytes in culture: fatal flaw or soluble problem? *Stem Cells Dev.* 24, 1035-1052. 10.1089/scd.2014.0533 25583389

[DMM037994C40] Veerman C. C., Mengarelli I., Guan K., Stauske M., Barc J., Tan H. L., Wilde A. AM., Verkerk A. O. and Bezzina C. R. (2016). hiPSC-derived cardiomyocytes from Brugada Syndrome patients without identified mutations do not exhibit clear cellular electrophysiological abnormalities. *Sci. Rep.* 6, 30967 10.1038/srep30967 27485484 PMC4971529

[DMM037994C41] Verkerk A. O. and Wilders R. (2015). Pacemaker activity of the human sinoatrial node: an update on the effects of mutations in *HCN4* on the hyperpolarization-activated current. *Int. J. Mol. Sci.* 16, 3071-3094. 10.3390/ijms16023071 25642760 PMC4346881

[DMM037994C42] Verkerk A. O., Veerman C. C., Zegers J. G., Mengarelli I., Bezzina C. R. and Wilders R. (2017). Patch-clamp recording from human induced pluripotent stem cell-derived cardiomyocytes: improving action potential characteristics through dynamic clamp. *Int. J. Mol. Sci.* 18, E1873 10.3390/ijms18091873 28867785 PMC5618522

[DMM037994C43] Vernon H., Cohen J., De Nittis P., Fatemi A., McClellan R., Goldstein A., Malerba N., Guex N., Reymond A. and Merla G. (2018). Intellectual developmental disorder with cardiac arrhythmia syndrome in a child with compound heterozygous GNB5 variants. *Clin. Genet.* 93, 1254-1256. 10.1111/cge.13194 29368331

[DMM037994C44] Westerfield M. (2000). *The Zebrafish Book. A Guide for the Laboratory use of Zebrafish (Danio rerio)*, Vol. Eugene, Oregon, USA: University of Oregon Press Eugene, Oregon, USA: University of Oregon Press.

[DMM037994C45] Wickman K. D., Iñiguez-Lluhl J. A., Davenport P. A., Taussig R., Krapivinsky G. B., Linder M. E., Gilman A. G. and Clapham D. E. (1994). Recombinant G-protein beta gamma-subunits activate the muscarinic-gated atrial potassium channel. *Nature* 368, 255-257. 10.1038/368255a0 8145826

[DMM037994C46] Wickman K., Nemec J., Gendler S. J. and Clapham D. E. (1998). Abnormal heart rate regulation in GIRK4 knockout mice. *Neuron* 20, 103-114. 10.1016/S0896-6273(00)80438-9 9459446

[DMM037994C47] Wydeven N., Posokhova E., Xia Z., Martemyanov K. A. and Wickman K. (2014). RGS6, but not RGS4, is the dominant regulator of G protein signaling (RGS) modulator of the parasympathetic regulation of mouse heart rate. *J. Biol. Chem.* 289, 2440-2449. 10.1074/jbc.M113.520742 24318880 PMC3900986

[DMM037994C48] Yang J., Huang J., Maity B., Gao Z., Lorca R. A., Gudmundsson H., Li J., Stewart A., Swaminathan P. D., Ibeawuchi S.-R. et al. (2010). RGS6, a modulator of parasympathetic activation in heart. *Circ. Res.* 107, 1345-1349. 10.1161/CIRCRESAHA.110.224220 20864673 PMC2997524

