# Supplementary material for: Genetic variation in GNB5 causes bradycardia by augmenting the cholinergic response via increased acetylcholine-activated potassium current (IK,ACh)
Source: Dis Model Mech. 2019 Jul 9;12(7):dmm037994. doi: 10.1242/dmm.037994 (PMC6679373; doi:10.1242/dmm.037994)
Supplement: Supplementary information [file dmm-12-037994-s1.pdf]

SUPPLEMENTARY INFORMATION

Supplementary Figures

Figure S1. Generation of genome-edited hiPSC lines. **A**, Using CRISPR/Cas9 technology, the c.242C>T p.S81L *GNB5* mutation was inserted into a control hiPSC line in heterozygous and homozygous state. **B**, Location of the c.242C>T mutation in the *GNB5* transcript and the surrounding sequence. The gRNA sequence that was used is indicated in blue, the PAM sequence in bold. In the single-stranded oligonucleotide (ssODN) that was applied for homologous recombination the c.242C>T mutation is indicated in red and bold, whereas red and italic indicates the synonymous mutation generating a restriction site used for colony screening. **C**, Sanger sequences of wildtype (upper panel), S81L heterozygous (middle panel) and S81L homozygous (lower panel) clones. **D and E**, Immunocytochemistry of the pluripotency markers OCT4, TRA1-81, NANOG, and SSEA4 in the S81L heterozygous (D) and homozygous (E) lines. Scale bars are 100 µm. **F and G**, Normal karyotypes exhibited by hiPSC lines S81L^{het} (F) (verified by COBRA-FISH) and S81L^{homo} (G) (verified by G banding).

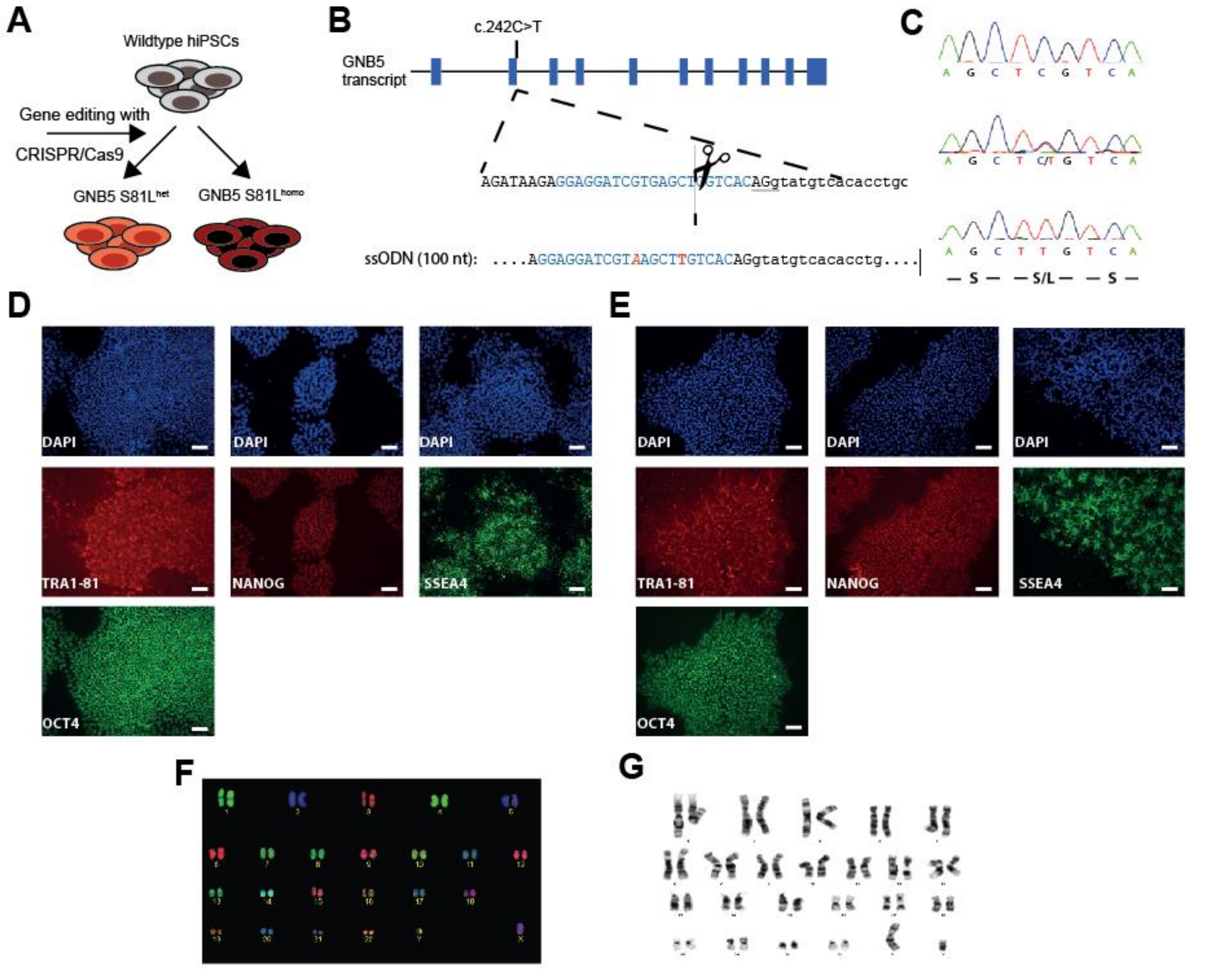

Disease Models & Mechanisms • Supplementary information

Figure S2. Action potential (AP) characteristics of retinoic acid (RA) and DMSO-treated hiPSC-CMs. A. Typical example of an action potential when paced at 1 Hz. **B.** Action potential duration at 20%, 50% and 80% of repolarization (APD_{20}, APD_{50} and APD_{80} respectively). In RA-treated hiPSC-CMs, APD is significantly shorter as compared to DMSO-treated hiPSC-CMs (**P*<0.05, two-sided t-test). **C.** Individual plateau amplitudes, demonstrating lower values in RA-treated hiPSC-CMs in comparison with DMSO-treated hiPSC-CMs (**P*<0.05, , two-sided t-test) . **D.** Upstroke velocity. **E.** Effects of carbachol (CCh) on maximal diastolic potential (MDP) in DMSO- and RA-treated hiPSC-CMs. While no effect on MDP is found in DMSO-treated hiPSC-CMs, RA-treated hiSPC-CMs display a significant hyperpolarization upon CCh application (**P*<0.05, paired t-test). B-E. DMSO, n=7; RA, n=17 hiPSC-CMs.

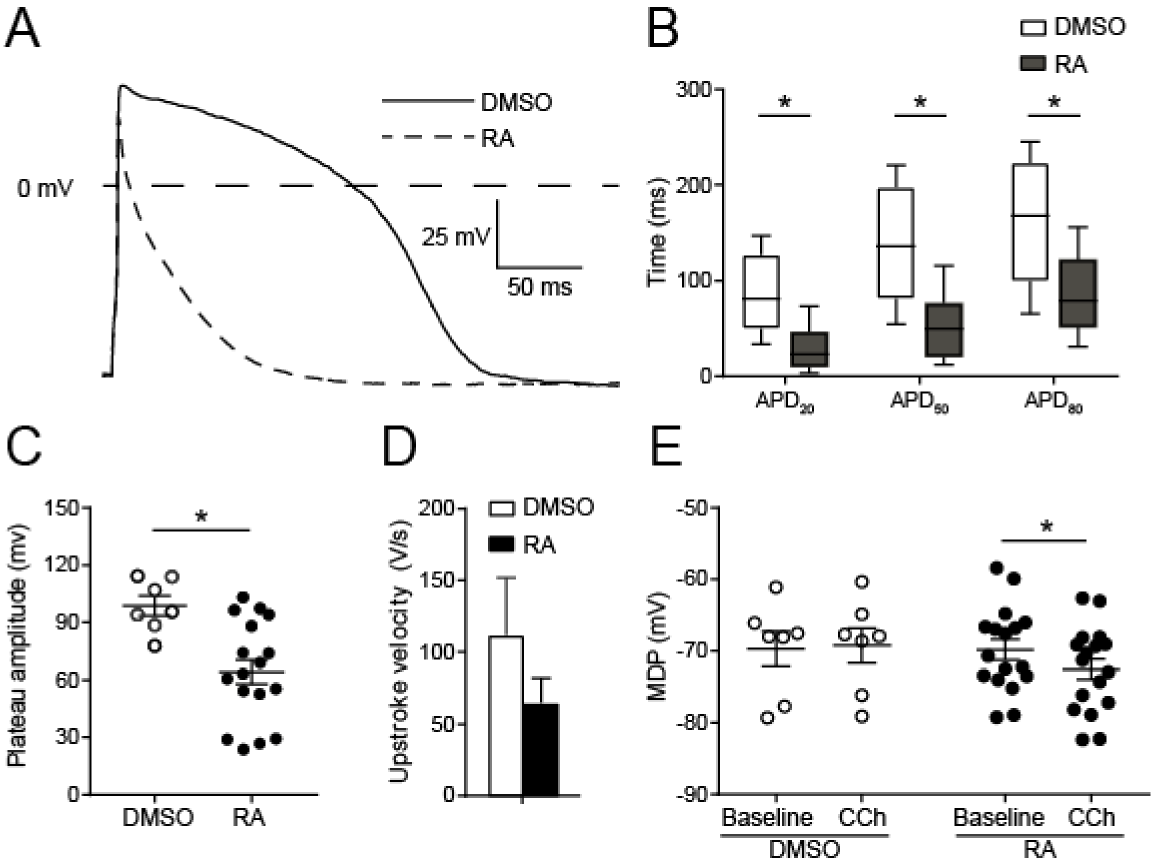

Disease Models & Mechanisms • Supplementary information

Figure S3. Expression level of genes *GNB1,2,4* and *TNNT2* as assessed by RT-qPCR in WT, S81L^{het} and S81L^{homo} hiPSC-CMs. Data for each gene are presented as mean+/-s.e.m. of three biological replicas and are normalized to the expression of *TBP*. Quantification is expressed as relative to the expression in the wild type (WT) clone.

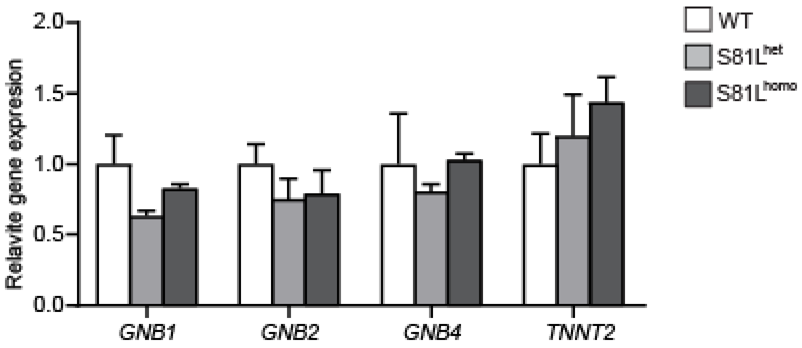

Supplementary Tables

Table S1. Primer sequences used for PCRs.

Gene	Forward 5' to 3'	Reverse 5' to 3'
GNB5-screen	AGCAAGGGGACTGACTATGG	AAACCCGCTCACCTGTTATG
TBP	GCTCACCCACCAACAATTTAG	TCTGCTCTGACTTTAGCACCTG
NR2F2	GCCATAGTCCTGTTCACCTCA	AATCTCGTCGGCTGGTTG
NPPA	GCTCCTTCTCCACCACCAC	GGGCACGACCTCATCTTCTA
KCNJ3	ACCTATCCCAGCGAAGCAT	AAAACGATGACCCCAAAGAA
KCNJ5	GAGCCCTTTCTGGGAGATGT	TTTCCAAGGTGAGGACTGGTG
GNB5	CGTGTCGCCTCTATGACCTG	CTTTGAGAACATCCCAGACG
RGS6	AAATCTGGCAAGAGTTTCTGG	GGGCATAGCTGTCACTCTTCA
GNB5-OT1	GGTCCCAGGAGAGTTTCTGT	GCTTTGAGAACGAGGGTGTC
GNB5-OT2	TGTGGCAAAAACACATAAACG	CAGCTCCTGGCCTTTGTTA
GNB5-OT3	CTCACCCCCAGCCTTAGA	AGAGGCTGGGCTTTTGCTAT
GNB1	GACCGGGCAGGTGTCTTG	GTTCCAGATCTTGAGGAAGCTA
GNB2[*]	AGATCACAGCTGGGCTG	GATGAGCTTCCCATCCTGG
GNB3	GATCTGCTTCTCCCACGAGAG	GATGCCCACACGCTCAGAC
GNB4	AAAAAGTGGGCGTCTCTTGTTG	CAGCTCACACGGTTGTCATGA
TNNT2	GACCAGGGCAGAAGAAGATG	ACTCTCTCTCCATCGGGGAT

*__Stallmeyer B, Kuß J, Kotthoff S, Zumhagen S, Vowinkel K, Rinné S, Matschke LA, Friedrich C, Schulze-Bahr E, Rust S, Seebohm G, Decher N, Schulze-Bahr E__.(2017). A Mutation in the G-Protein Gene *GNB2* Causes Familial Sinus Node and Atrioventricular Conduction Dysfunction. Circ. Res. 120(10):e33-e44.

Disease Models & Mechanisms • Supplementary information

Table S2. Antibodies used for immunocitochemistry.

Primary Antibodies					
**Antigen**	**Isotype**	**Supplier**	**Cat. Number**	**Dilution**	**Citation (PMID)**
OCT3/4	Mouse IgG2b	Santa Cruz Biotechnology	sc-5279	1:100	30778227
TRA 1-81	Mouse IgM	Abcam	ab 16289	1:67	28344003
SSEA-4	Mouse Ig3	Santa Cruz Biotechnology	sc-59368	1:100	29030611
NANOG	Goat IgG	R&D Systems	AF 1997	1:33	23910086
**Secondary Antibodies**					
**Host species**	**Antigen**	**Supplier**	**Cat. Number**	**Dilution**	**Fluorophore**
Goat	Anti-mouse IgG2b	ThermoFisher Scientific	A-21141	1:500	AF488
Goat	Anti-mouse IgM	ThermoFisher Scientific	A-21426	1:500	AF555
Goat	Anti-mouse $IgG_{(H+L)}$	ThermoFisher Scientific	A-11001	1:500	AF488
Donkey	Anti-goat $IgG_{(H+L)}$	ThermoFisher Scientific	A-11057	1:500	AF568

Disease Models & Mechanisms • Supplementary information

Table S3. Putative genomic off-target sites for the gRNA used to introduce mutation p.S81L in *GNB5*.

Location	Genomic sequence	Mismatches	Strand	Type
15:52153939-52153961	GAGGATCGTGAGCTCGTCAC *AGG*			
22:19979742-19979764	GAGG**TC**CGTGAGCT**T**GTCAC *TGG*	3	–	Exonic
16:77913475-77913497	GAGGATCG**A**GAGCT**GA**TCAC *TGG*	3	–	Intronic
1:160408628-160408650	GAGGAT**G**GTGAGCTC**A**TC**T**C *TGG*	3	+	Intronic

Original gRNA: first row. Sequence in italics depicts the PAM site (NGG), in bold and red the mismatches with the original gRNA.

Disease Models & Mechanisms • Supplementary information

Table S4. Action potential (AP) parameters in WT, S81L^{het} and S81L^{homo} at baseline in spontaneously fast beating (>1 Hz) hiPSC-CMs.

	WT (n=12)	S81L^{het} (n=10)	S81L^{homo} (n=13)
MDP (mV)	−68.5±0.6	−69.3±1.3	−70.2±1.8
APA (mV)	94.7±4.6	86.5±4.9	85.6±4.9
V_{max} (V/s)	40.9±9.9	28.3±8.2	32.9±8.9
APD_{20} (ms)	45.3±5.7	49.4±12.7	39.0±4.4
APD_{50} (ms)	81.5±9.7	82.4±19.2	66.6±6.5
APD_{80} (ms)	109.0±12.6	103.8±19.5	88.8±8.4
$APA_{plateau}$ (mV)	86.1±4.8	75.0±7.7	77.9±5.5

Data are mean±s.e.m., MDP, maximal diastolic potential; APA, AP amplitude; V_{max}, maximal upstroke velocity; APD_{20}, APD_{50}, APD_{80}, AP duration at 20, 50, and 80% of repolarization, respectively; $APA_{plateau}$, AP plateau amplitude.
